# Selective autophagy fine-tunes Stat92E activity by degrading Su(var)2-10/PIAS in *Drosophila* glia

**DOI:** 10.26508/lsa.202503375

**Published:** 2026-01-07

**Authors:** Virág Vincze, Zsombor Esküdt, Erzsébet Fehér-Juhász, Aishwarya Sanjay Chhatre, András Jipa, Anna Rita Galambos, Dalma Feil-Börcsök, Melinda Bence, Gábor Juhász, Áron Szabó

**Affiliations:** 1 HUN-REN Biological Research Center, Institute of Genetics, Szeged, Hungary; 2 Doctoral School of Biology, University of Szeged, Szeged, Hungary; 3 National Academy of Scientist Education Program of the National Biomedical Foundation, Szeged, Hungary; 4 Albert Szent-Györgyi Medical School, University of Szeged, Szeged, Hungary; 5 Department of Anatomy, Cell and Developmental Biology, Eötvös Loránd University, Budapest, Hungary

## Abstract

Glial immunity plays a pivotal role in the maintenance of nervous system homeostasis and in responses to stress conditions, including neural injuries. The transcription factor Stat92E is activated independently of the canonical JAK/STAT pathway in *Drosophila* glial cells after brain injury to shape glial reactivity toward degenerating axons. However, the upstream regulatory mechanisms governing Stat92E activation remain elusive. Here, we reveal that selective autophagy gates nuclear translocation of Stat92E after injury and directs the degradation of the PIAS SUMO ligase family member Stat92E repressor, Su(var)2-10, in glia. Autophagic elimination of Su(var)2-10 mediated by its colocalization and interaction with the core autophagy factor Atg8a is required for efficient Stat92E-dependent transcription after injury. In line with this, we demonstrate that autophagy is essential for the up-regulation of an innate immune pathway in glial cells after axon injury, characterized by the induction of *virus-induced RNA 1* (*vir-1*). We propose that autophagic Su(var)2-10 breakdown controls Stat92E activation to allow glial reactivity. These findings identify a critical role of autophagy in glial immunity as part of nervous system injury responses.

## Abstract (precis)

The study shows that glial Stat92E activity is derepressed by selective autophagic elimination of the Stat92E repressor Su(var)2-10/PIAS in the injured *Drosophila* wing nerve.

## Graphical Abstract

**Figure:**
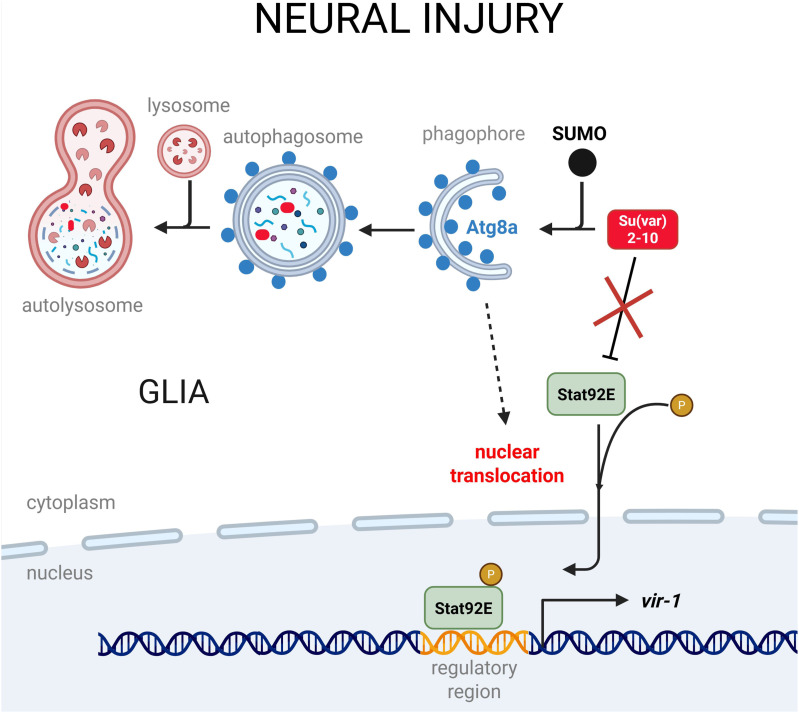

## Introduction

The brain tissue in homeostatic conditions is constantly monitored by glia to mount an enhanced response in the face of a challenge. Microglia persistently sample the environment: their elongated processes search for signals emitted from dying, damaged, or infected cells. These trigger morphological and signaling changes in microglia to induce a transformation into a reactive state. Astrocytes undergo a similar transition upon stimuli. Reactive glia have distinct transcriptional signatures as exemplified by disease-associated microglia in neurodegeneration (Zengeler & Lukens, 2024). They sense damage- and pathogen-associated molecular patterns (DAMPs and PAMPs, respectively), also known as find-me and eat-me cues via dedicated receptors to mount an immune response. They also release various chemokines and cytokines to control neuroinflammation (Younger et al, 2019). Upon central nervous system (CNS) injury, these receptors trigger signaling events that involve the MAPK, JAK-STAT, and NF-κB pathways (Younger et al, 2019). The transcriptomics changes in reactive glia show a major contribution from these signaling networks (Dominguez et al, 2008; Herrmann et al, 2008; Zhao et al, 2011; Zamanian et al, 2012; Balak et al, 2024).

The JAK-STAT pathway is an important but incompletely characterized arm of glial immunity, especially when compared to the NF-κB pathway (Yan et al, 2018). Most interleukin and interferon receptors signal through JAK-STAT in mammals and induce the expression of either pro- or anti-inflammatory cytokines depending on the STAT paralog involved, both in peripheral immune cells and in glia (Yan et al, 2018; Jain et al, 2021). In neurodegenerative diseases, the JAK-STAT pathway contributes to neuroinflammation. Increased interferon-dependent microglial STAT1 activation is observed in models of Alzheimer’s disease, causing complement-dependent synapse elimination (Roy et al, 2020).

Research on *Drosophila* has elucidated key early glial responses to axon injury (MacDonald et al, 2006, 2013; Ziegenfuss et al, 2008, 2012; Lu et al, 2017). The single STAT family transcription factor Stat92E up-regulates the main glial phagocytic receptor *draper* (*drpr*) and matrix metalloproteinase *Mmp1* to induce glial reactivity (Doherty et al, 2014; Purice et al, 2017). Interestingly, this response does not rely on canonical pathway components such as JAK kinase (Hopscotch/Hop in flies) or its upstream receptor Domeless (Dome) (Doherty et al, 2014). Instead, the Drpr receptor and its downstream signaling partners involved in Rac1 activation are required for glial Stat92E activation after injury (Doherty et al, 2014). Thus, Drpr binding to its ligand initiates a positive feedback loop in glial cells to promote *drpr* transcription. Insulin-like signaling also promotes glial Stat92E activity (Musashe et al, 2016). How Rac1 relays signaling to Stat92E is not known (Doherty et al, 2014). However, besides Stat92E, the transcription factor AP-1 also promotes *drpr* and *Mmp1* up-regulation in glia after injury (MacDonald et al, 2013; Purice et al, 2017).

Macroautophagy (simply referred to as autophagy) is a membrane-limited intracellular degradation pathway that removes cytoplasmic components including protein aggregates and organelles (Mulakkal et al, 2014). Upon induction by starvation or other stress conditions, an Atg1 (ULK1/2) complex initiates the formation of autophagic membranes. A network of Atg proteins mediates the formation of a cup-shaped phagophore that gives rise to an autophagosome, which transports cargo to lysosomes. This process requires Atg8a lipidation via two ubiquitin-like conjugation systems: Atg8a gets lipidated and thus membrane-bound by the Atg16/Atg5-Atg12 complex. Autophagosomes fuse with lysosomes where cargo breakdown and recycling take place.

A targeted variation on this theme is selective autophagy. During this process, cargo receptors recognize specific cellular components ranging from organelles to single molecules and recruit the autophagosome biogenesis machinery irrespective of nutritional status (Gohel et al, 2020; Adriaenssens et al, 2022). Cargo receptors can bind to ubiquitylated material or they can be embedded in target organelles, but labile proteins can also feature receptor-like motifs that mediate direct degradation (Gohel et al, 2020; Adriaenssens et al, 2022).

Because Stat92E activation is pivotal to glial adaptation to challenges, we need to gain a better insight into its regulatory mechanisms. Here, we show that autophagy clears the direct Stat92E repressor PIAS/Su(var)2-10 in wing glia after wing transection, thereby gating Stat92E-dependent transcriptional regulation. Interestingly, *drpr* expression is not responsive to injury in these peripheral glial types. We find that another Stat92E target, *vir-1*, is induced in an autophagy-dependent manner after wing transection.

## Results

### Selective autophagy is necessary for Stat92E but not for JNK pathway activation

**Figure S1.**
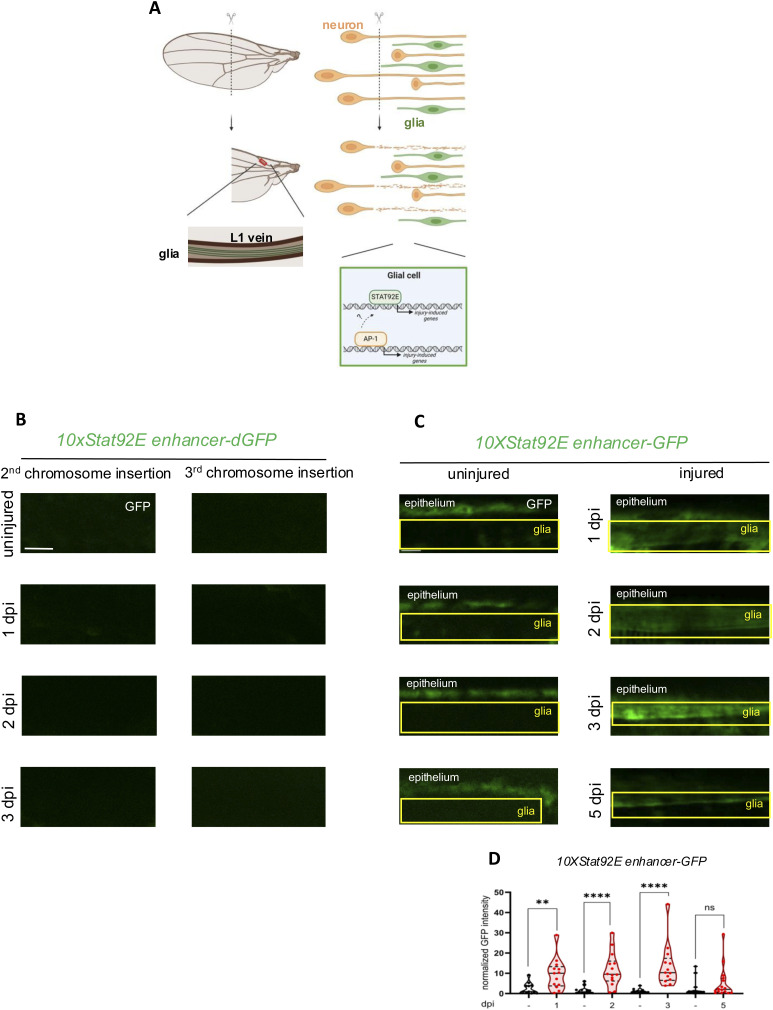
Stat92E and AP-1 transcriptional reporters are activated in wing glia after injury. **(A)** Schematic representation of the injury-induced glial reactivity in the *Drosophila* wing. After transecting the wing, severed axons (ochre) will undergo Wallerian degeneration and trigger reactivity in circumambient glia (green). This results in transcriptional activation by signaling pathways. It is known that transcription factors AP-1 of the JNK pathway and Stat92E are activated after axonal injury in CNS glia. The rectangular-shaped outline indicates the imaged area within the L1 vein nerve. **(B)** Single-slice images of wing veins from two independent insertion lines of *10xStat92E enhancer*-*dGFP* 1, 2, and 3 dpi. Please note the lack of detectable dGFP expression. Scale bar: 10 μm. **(C)** Single-slice images of wings with *10xStat92E enhancer-GFP* signal in glia 1, 2, 3, and 5 dpi show the dynamic activity of the Stat92E transcription factor. Scale bar: 5 μm. **(D)** Quantitative analysis of *10xStat92E enhancer-GFP* signal intensity at four different time points shown in (C). Black—uninjured; red—injured cohorts. Truncated violin plots are shown with median and quartiles. An unpaired, two-tailed Mann–Whitney test was used for statistics. ****P < 0.0001, **P = 0.0024, ns = 0.1627. n = 12, 13, 13, 14, 13, 13, 15, 16. The boxed area indicates the position of wing nerve glia adjacent to epithelial GFP signal.

**Figure 1.**
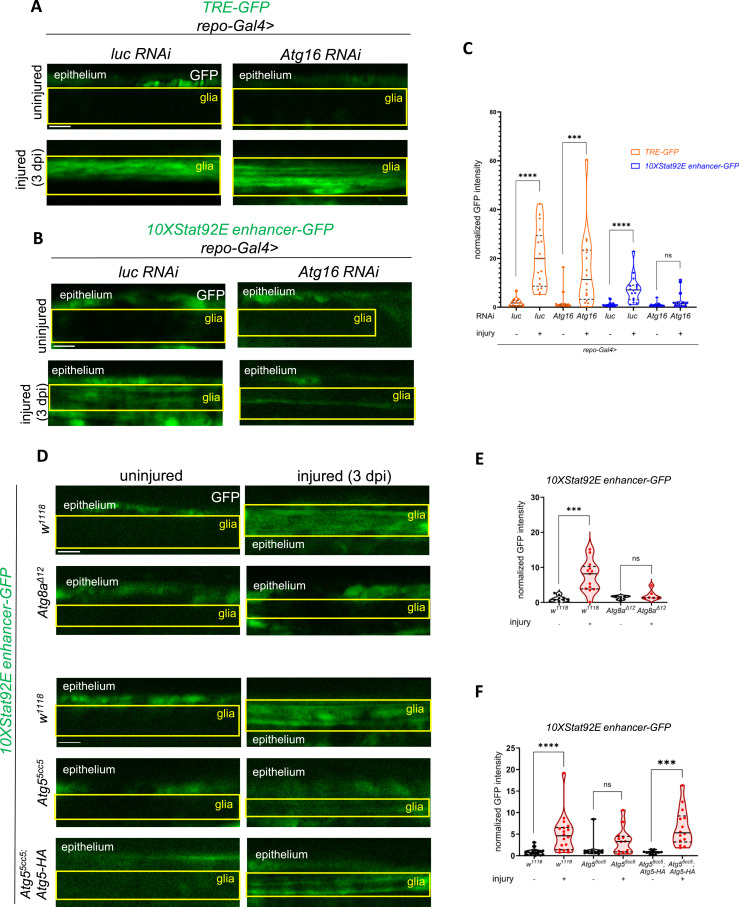
*Atg16* is required for Stat92E-dependent, but not for AP-1–dependent transcription during glial reactivity. **(A, B)** Single-slice images showing the effect of *Atg16* and control (firefly luciferase: *luc*) knockdowns in glia on the *TRE-EGFP* and *10xStat92E enhancer-GFP* reporters in uninjured and injured wing nerves at 3 dpi (days post-injury; see also Fig S1A). RNAi expression was induced by glia-specific *repo-Gal4*. **(C)** Quantitative analysis of the activity of the reporter constructs in (A, B) based on normalized GFP intensity. An unpaired, two-tailed Mann–Whitney test was applied in each comparison. ****P < 0.0001, ***P = 0.0004, ns = 0.0743. n = 13, 17, 13, 16, 18, 16, 16, 17. **(D)** Single-slice images of wing nerves showing the *10xStat92E enhancer-GFP* signal at 3 dpi in *Atg8a*^*Δ12*^ and *Atg5*^*5cc5*^ mutant backgrounds. The *Atg5*^*5cc5*^ phenotype can be rescued by introducing *Atg5::3xHA*, an endogenous promoter-driven *Atg5* transgene. **(E, F)** Quantitative analysis of *10xStat92E enhancer-GFP* activation based on normalized GFP intensity. Unpaired, two-tailed *t* test (*w*^*1118*^ (E), *Atg5*^*5cc5*^*::3xHA*) and unpaired, two-tailed Mann–Whitney test (*w*^*1118*^ (F), *Atg8a*^*Δ12*^, *Atg5*^5cc5^) were used for statistics. ****P < 0.0001, ***P = 0.0002, ns (E) > 0.9999, ns (F) = 0.1179. n (E) = 10, 13, 4, 6. n (F) = 17, 18, 11, 12, 11, 14. Truncated violin plots are shown with median and quartiles. The boxed area indicates the position of wing nerve glia adjacent to epithelial GFP signal. Scale bar: 5 μm. Source data are available for this figure.

To better understand nervous system adaptation after injury, we investigated whether autophagy impacts signaling pathways in glia. Two well-characterized immune pathways in glial responses are the JAK-STAT and JNK pathways. We used a well-established *Drosophila* wing injury model (Fang et al, 2012; Neukomm et al, 2014) (Fig S1A) to measure transgenic reporter up-regulation for either of these pathways. *TRE-EGFP* (Chatterjee & Bohmann, 2012) harbors four copies of an artificial tetradecanoylphorbol acetate response element (TRE) as an optimal AP-1–binding site fused upstream of a basal promoter and EGFP. The commonly used reporter *10xStat92E-GFP* (Bach et al, 2007) is composed of five copies of *Socs36E* intron 1 enhancer element, each of which contains at least two Stat92E-binding sites, and these are fused to a *hsp70* minimal promoter and EGFP to detect enhancer activity. To avoid confusion with a *Stat92E::GFP* protein–protein fusion transgene, we will refer to this reporter as *10xStat92E enhancer-GFP* hereafter. Similar to the antennal lobe in the central nervous system (CNS) after antennal ablation (MacDonald et al, 2013; Doherty et al, 2014; Lu et al, 2017), both the AP-1 transcriptional reporter and the Stat92E transcriptional reporter showed robust activation after wing nerve injury (Fig 1A and B). *10xStat92E enhancer*-*destabilized (d)GFP* reflects gene expression changes more dynamically compared with *10xStat92E enhancer-GFP* (Bach et al, 2007), dGFP half-life being 2 versus 24 h of EGFP. However, we failed to find detectable expression from *10xStat92E enhancer*-*dGFP* in the wing nerve 0, 1, 2, and 3 d post-injury (Fig S1B). Therefore, we set out to establish the dynamics of *10xStat92E enhancer-GFP* up-regulation in the wing nerve. We first followed GFP reporter intensity at different time points after injury (Fig S1C and D). *10xStat92E enhancer-GFP* signal was undetected in the wing nerve of uninjured animals, and its intensity peaked at 3 d post-injury (3 dpi) with a decline at 5 dpi; therefore, we subsequently measured reporter activation levels at 3 dpi. These results also indicate that *10xStat92E enhancer-GFP* expression is dynamic after injury in the wing nerve unlike in the brain where this reporter is well expressed in uninjured conditions and hardly increases after antennal ablation (Doherty et al, 2014).

**Figure S2.**
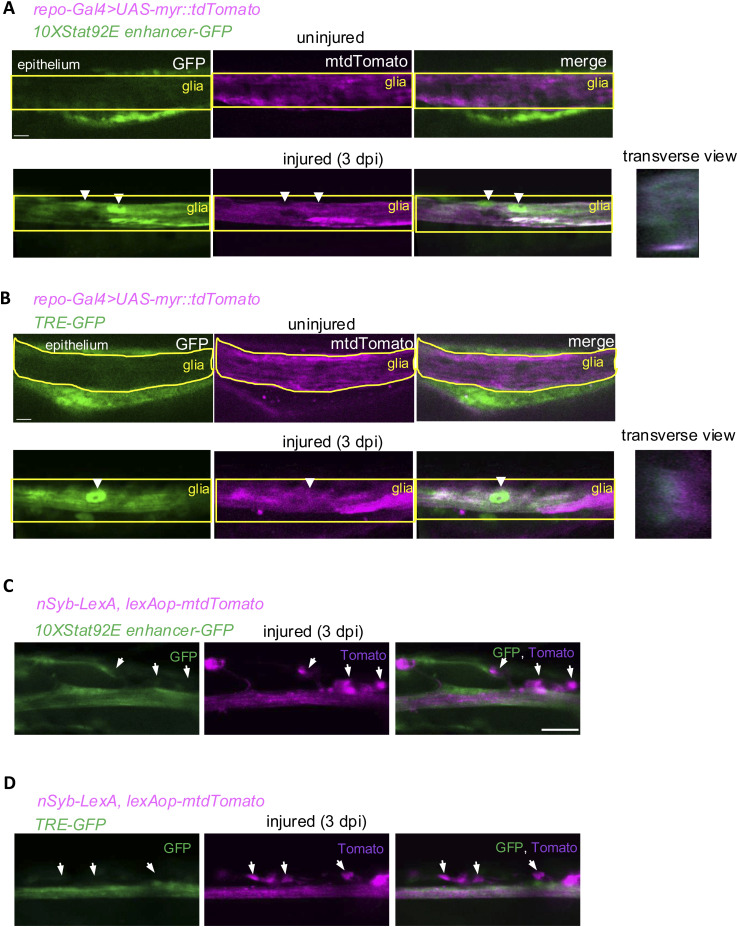
*10xStat92E enhancer-GFP* and *TRE-EGFP* are expressed in glia but not in neurons in the wing nerve. **(A, B)** Fluorescent microscopic images of uninjured and injured wing nerves 3 dpi. Single longitudinal optical slices are shown in each case with a single transversal slice for the injured specimens. *repo-Gal4*–driven myr::mtdTomato colocalizes with the signal from the *10xStat92E enhancer-GFP* and the *TRE-EGFP* reporters in glia. Please note that the elongated fibers that are outlined by reporter expression could potentially correspond to either glia or axons. Glia, in the wing nerve and generally in the peripheral nervous system, especially wrapping glia, have a filament-like appearance that can resemble axons (Neukomm et al, 2014). Arrowheads denote glial nuclei. Scale bar: 5 μm. **(C, D)** Fluorescent microscopic images of neuronal cell bodies of the wing nerve in injured anterior wing margins at 3 dpi. Arrows point to neuronal cell bodies, which occupy a stereotypical position at the base of the sensory bristles at the sensilla. Single optical slices are shown in each case. Signal of the *nSyb-LexA*–driven mtdTomato^+^ cell bodies does not colocalize with the signal from the *10xStat92E enhancer-GFP* and the *TRE-EGFP* reporters in neuronal cell bodies. Neuronal expression of *TRE-EGFP* and *10xStat92E enhancer-GFP* therefore can be excluded because these reporters do not outline the wing vein neuronal somata. Scale bar: 10 μm.

The expression pattern of both *10xStat92E enhancer-GFP* and *TRE-EGFP* coincided with a myr::tdTomato signal expressed in glia (Fig S2A and B), similar to the glial up-regulation of these reporters in the CNS. In contrast, reporter signal did not colocalize with neuronal cell bodies in the wing margin, indicating that injury triggers glial rather than neuronal expression of *10xStat92E enhancer-GFP* and *TRE-EGFP* (Fig S2C), in line with previous reports (MacDonald et al, 2013; Doherty et al, 2014).

**Figure S3.**
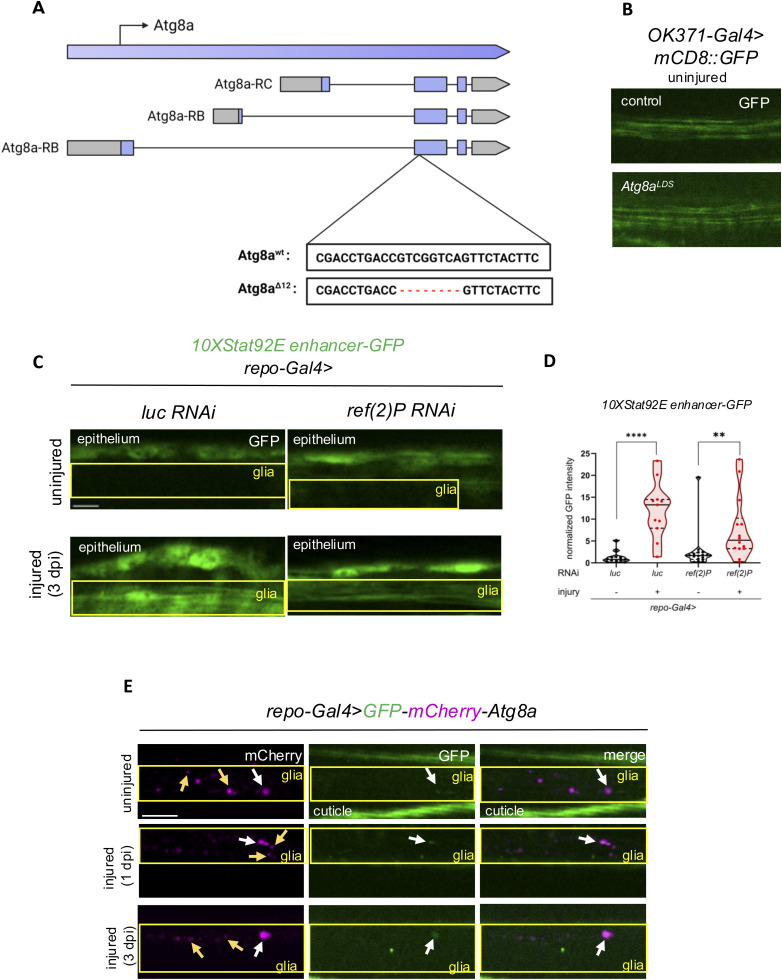
Ref(2)P/p62 cargo receptor is not involved in Stat92E activation and the volume of bulk autophagy is not increased in glia after wing injury. **(A)** Schematic illustration shows the site and sequence of the mutation in the CRISPR/Cas9-generated *Atg8a*^*Δ12*^ flies, which causes a frameshift in all transcript variants. **(B)** Single optical slices of wing nerves with *OK371-Gal4, UAS-mCD8::GFP*-labeled axons in the indicated genotypes. **(C)** Single optical slices of wing nerves with glial *ref(2)P* silencing in a *10xStat92E enhancer-GFP* background. RNAi expression was induced by *repo-Gal4*. The boxed area indicates the position of wing nerve glia adjacent to epithelial GFP signal. Scale bar: 5 μm. **(D)** Quantitative analysis of *10xStat92E enhancer-GFP* signal in *ref(2)P* RNAi. Truncated violin plots are shown with median and quartiles. The analyses were carried out by an unpaired, two-tailed Mann–Whitney test. ****P < 0.0001. **P = 0.0013. n = 12, 11, 16, 14. **(E)** Wings of *repo-Gal4>UAS-GFP-mCherry-Atg8a* flies were transected and imaged at the indicated time points. White arrows point to double GFP+ mCherry+ vesicles, whereas yellow arrows point to only mCherry+ vesicles. mCherry is stably fluorescent in lysosomes, whereas GFP is rapidly quenched; therefore, autolysosomes are exclusively mCherry+, whereas other autophagic structures are doubly labeled. The boxed area indicates the position of wing nerve glia adjacent to cuticular autofluorescence. Scale bar: 10 μm.

We then depleted the core autophagy factor *Atg16* specifically in glia using *repo-Gal4*. Although *Atg16* silencing did not affect *TRE-EGFP* expression, it abrogated *10xStat92E enhancer-GFP* expression after injury (Fig 1A–C). We also measured Stat92E reporter activity in *Atg5 (Atg5*^*5cc5*^*)* and *Atg8a (Atg8a*^*Δ12*^*)* null mutant (Fig S3A) backgrounds and observed a comparable effect on *Atg16* knockdown (Fig 1D–F). Importantly, lack of Stat92E activation could be rescued by an endogenous promoter-driven *Atg5* transgene (*Atg5::3xHA*) in the *Atg5*-deficient background (Fig 1D and F).

**Figure 2.**
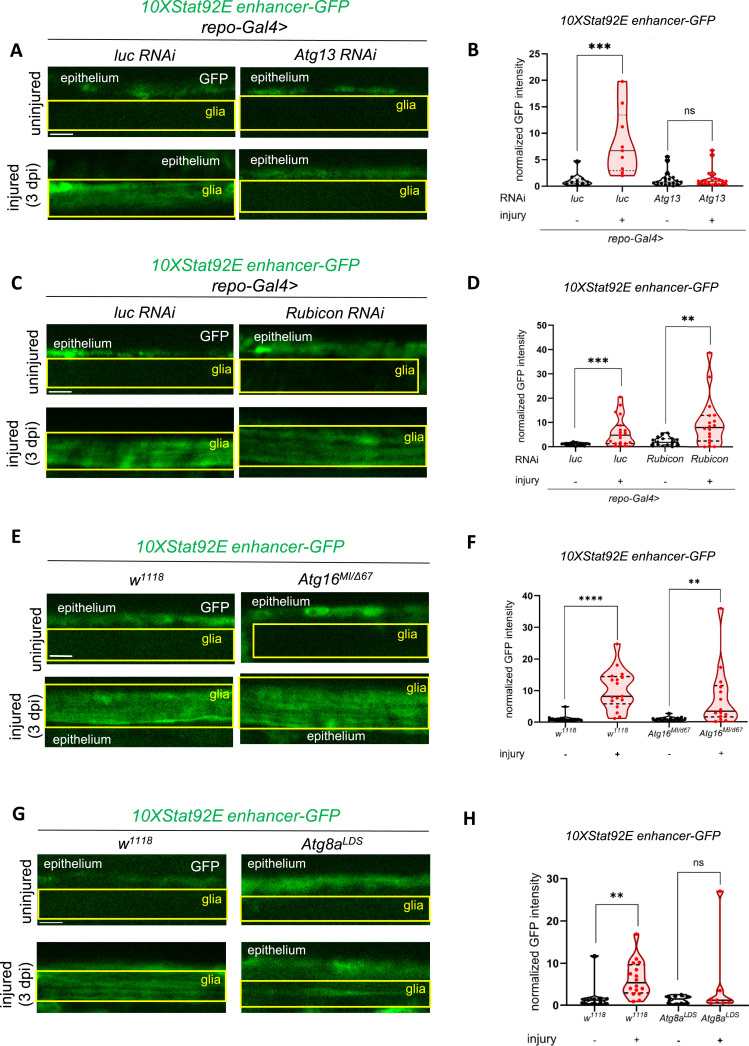
Selective autophagy drives Stat92E-dependent transcription in injury signaling. **(A, C)** Single-slice images of wing nerves showing *10xStat92E enhancer-GFP* signal in different glial RNAi backgrounds. *Atg13* is required for autophagy, whereas *Rubicon* is indispensable for LAP (LC3/Atg8a-associated phagocytosis). RNAi expression was induced by *repo-Gal4*. **(B, D)** Quantitative analysis of *10xStat92E enhancer-GFP* activation (A, C) based on normalized GFP intensity. An unpaired, two-tailed Mann–Whitney test was used for statistics. ***P (B) = 0.0002, ***P (D) = 0.0004, **P = 0.0021, ns = 0.9195. n (B) = 10, 9, 18, 20. n (D) = 19, 19, 18, 18. **(E, G)** Single optical slices showing the effect of the *Atg8a*^*LDS*^ mutation and *Atg16* WD40 repeat deficiency on *10xStat92E enhancer-GFP* activation. *Atg8a*^*LDS*^ has a mutated LIR docking site (LDS), which specifically eliminates LIR-dependent selective autophagy. *Atg16*^*M/IΔd67*^ combines Atg16 WD40 repeat disruption (*Atg16*^*MI*^) with an Atg16 null mutant (*Atg16*^*Δ67*^) in a transheterozygous state that impairs LAP. **(F, H)** Quantitative analysis of *10xStat92E enhancer-GFP* activity (from (E, G)) based on normalized GFP intensity. An unpaired, two-tailed Mann–Whitney test was used for each comparison. **(F)** ****P < 0.0001, **P = 0.0035, n = 15, 17, 14, 14, (H) **P = 0.0053, ns = 0.7789. n = 9, 16, 8, 7. Truncated violin plots are shown with median and quartiles. The boxed area indicates the position of wing nerve glia adjacent to epithelial GFP signal. Scale bar: 5 μm. Source data are available for this figure.

Atg5, Atg8a, and Atg16 are involved not only in autophagy but also in autophagy-related processes including LC3-associated phagocytosis (LAP) (Table S1), which we have recently characterized in glia in relation to axon debris degradation (Szabó et al, 2023). Therefore, we set out to distinguish the contribution of these two processes to Stat92E signaling. Atg13 is a key subunit of the autophagy-initiating Atg1 kinase complex, which does not participate in LAP. *Atg13* silencing in glia also abrogated Stat92E reporter activation (Fig 2A and B). Vice versa, Rubicon is dispensable for autophagy, but it is required for LC3-associated phagocytosis. Unlike *Atg13* knockdown, *Rubicon* RNA interference (RNAi) did not prevent Stat92E activation (Fig 2C and D). Furthermore, loss of single membrane Atg8a conjugation activity of Atg16 (*Atg16*^*MI/Δ67*^) that impairs LAP but not autophagy (Szabó et al, 2023) failed to affect Stat92E reporter up-regulation (Fig 2E and F). We thus conclude that canonical autophagy but not LAP sustains Stat92E signaling in glia.

Table S1. List of studied autophagy genes and their functions.

We aimed to further understand how autophagy specifically impacts Stat92E activation. Selective autophagy receptors (SARs) use their LC3-interacting region (LIR) to bind the LIR motif docking site (LDS) on the core autophagy protein Atg8a. SARs directly interact either with to-be-degraded cargos or with polyubiquitin (Ub) chains on cargos. LIRs can be also present in cargos themselves to facilitate their degradation via Atg8a binding. We thus evaluated a point mutant form of Atg8a that specifically disrupts the LDS to see whether it influences Stat92E-dependent gene activation (Rahman et al, 2022). Stat92E reporter up-regulation after injury was indeed impaired in homozygous *Atg8a*^*K48A/Y49A*^ *(Atg8a*^*LDS*^*)* mutant animals (Fig 2G and H). Of note, the *Atg8a*^*LDS*^ mutation did not affect axon morphology, similar to other *Atg* gene mutants (Szabó et al, 2023) (Fig S3B). Although silencing *ref(2)p/p62*, the only known ubiquitin chain–binding SAR in *Drosophila*, also showed a mild decrease in GFP reporter intensity, it did not have a statistically significant effect on *10xStat92E enhancer-GFP* activation (Fig S3C and D). Moreover, bulk autophagic degradation in glia did not change in response to wing nerve injury based on the tandem tagged autophagic flux reporter GFP-mCherry-Atg8a expressed in glia (Fig S3E) (Nagy et al, 2015). Here, the reporter exploits that Atg8a is anchored into forming autophagic membranes, and it is continuously delivered to lysosomes via autophagosomes. The GFP-mCherry-Atg8a reporter loses its green fluorescence in acidic lysosomes, whereas acid-resistant mCherry persists in lysosomes. Thus, degradative autolysosomes appear red because of quenching of GFP at their low internal pH, whereas initial autophagic structures such as autophagosomes are labeled by both GFP and mCherry. This allows the monitoring of bulk autophagic degradation (also known as flux) in a cell. These data suggest that selective autophagy is involved in Stat92E-dependent transcriptional activation in glia.

### *vir-1* but not *drpr* is up-regulated in response to autophagy-activated Stat92E signaling in glia after wing injury

**Figure S4.**
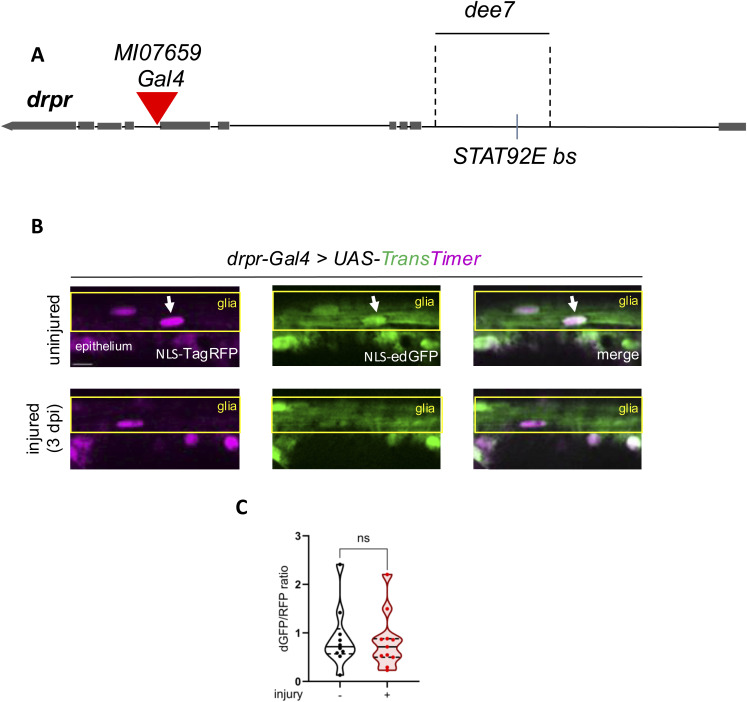
A *drpr* transcriptional reporter is not induced by injury in wing glia. **(A)** Schematic representation of the *drpr* locus. *dee7* bears a Stat92E-responsive enhancer including a functional Stat92E-binding site (bs). *MI07659* Trojan Gal4 conversion reflects endogenous *drpr* expression. **(B)** *drpr-Gal4* drives TransTimer (NLS-TagRFP and NLS-ed [enhanced destabilized] GFP separated by a 2A sequence) in the wing nerve. At 3 dpi, no major change is observed in the number of double GFP+RFP+ nuclei that reflects *drpr* promoter–enhancer activity. The arrows point to the nuclei. The boxed area indicates the position of wing nerve glia adjacent to epithelial GFP signal. Scale bar: 5 μm. **(C)** Truncated violin plots with median and quartiles are shown for the quantification of the NLS-ed GFP/NLS-TagRFP ratio in nuclei in *drpr-Gal4*–driven *UAS-TransTimer* flies without injury and 3 dpi. The analysis was carried out by an unpaired, two-tailed Mann–Whitney test. ns = 0.8094. n = 10, 11.

**Figure S5.**
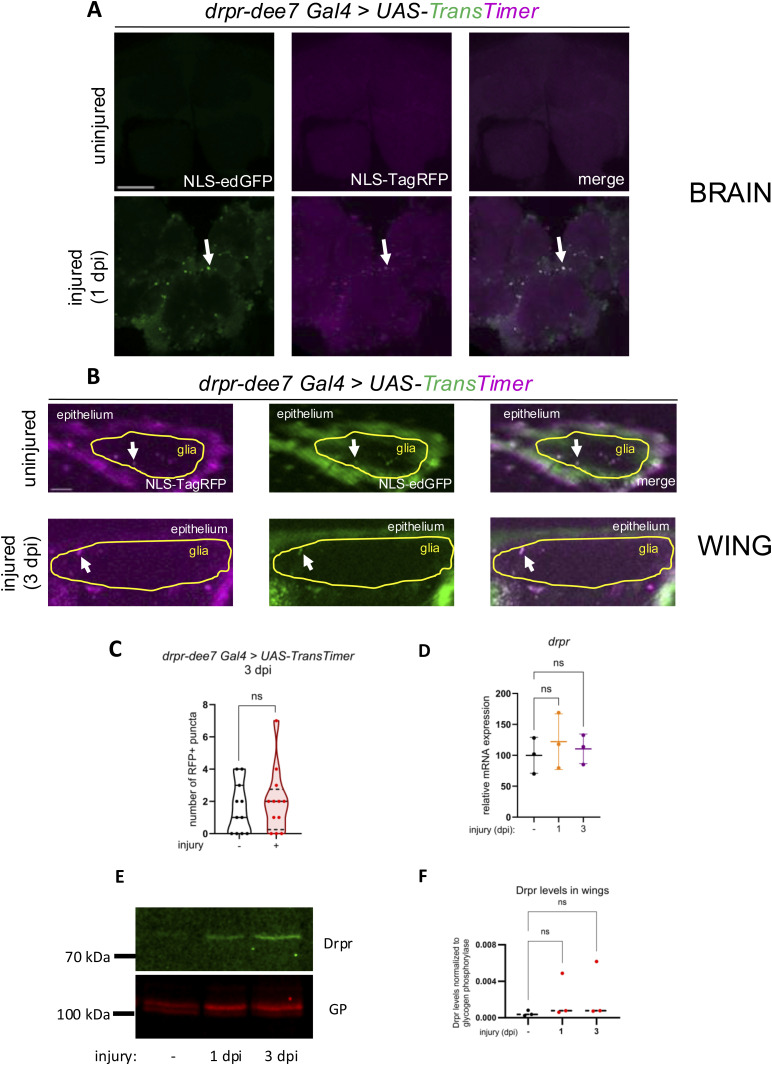
*drpr* enhancer (*dee7*) activity, *drpr* transcript and protein expression are not induced by injury in wing glia. **(A)** *dee7-Gal4* drives *UAS-TransTimer* in the brain. The central brain is shown 1 d after antennal ablation. In accordance with the published results (Doherty et al, 2014), *dee7* enhancer activity robustly increases after injury reflected by the appearance of double GFP+RFP+ nuclei. The arrows denote the RFP+ GFP+ puncta. Scale bar: 50 μm. **(B)** *dee7-Gal4* drives *UAS-TransTimer* in the wing nerve in uninjured and injured condition. As opposed to the brain, there is a basal activity of *dee7* in the wing nerve that does not appear to increase after wing transection. The arrows point to the RFP+ and/or GFP+ puncta. The boxed area indicates the position of wing nerve glia adjacent to epithelial GFP signal. Scale bar: 5 μm. **(C)** Statistical analysis of the number of RFP+ puncta in uninjured wings and at 3 dpi, shown in (B). Truncated violin plots with median and quartiles are shown. Statistics: unpaired, two-tailed Mann-Whitney test. ns = 0.6575. n = 11, 12. **(D)** Transcript levels of *drpr* in *w*^*1118*^ WT wings without injury and 1 and 3 d post-injury (dpi) determined by quantitative (q)PCR. *drpr* expression levels are normalized to *RpL32* expression. ns, not significant (*P* = 0.7030 and 0.7173, respectively). **(E)** Drpr protein levels by Western blot analysis of extracts from *w*^*1118*^ WT wings without injury and at 1 and 3 dpi. Glycogen phosphorylase (GP) serves as a loading control. **(F)** Quantification of band intensity ratios (Drpr/glycogen phosphorylase [GP]) from Western blots as in (E). ns, not significant. **(D, F)** Shown is the mean with SD; one-way ANOVA with the Holm–Šídák test for multiple comparisons correction was used for statistics.

What are the relevant targets of autophagy-regulated Stat92E activation in glial injury responses? After antennal ablation, baseline *drpr* expression in the brain increases via Stat92E-dependent transcription in response to Drpr receptor signaling, creating a positive feedback loop (Doherty et al, 2014). This is required for the efficient degradation of axon debris generated after injury. We monitored *drpr* transcription after neural injury to see whether it is under the influence of autophagic regulation. For this, we converted a MiMIC transposon insertion in *drpr* (*drpr*^MI07659^) into a *Trojan-Gal4*, in which Gal4 expression reflects *drpr* transcription (Fig S4A). We then expressed the recently developed transcriptional reporter *UAS-TransTimer* (He et al, 2019) with our *drpr-Gal4*. TransTimer allows measuring the ratio of destabilized nuclear GFP versus a stable nuclear RFP to monitor dynamic changes in transcription. Surprisingly, *drpr-Gal4* flies did not show *TransTimer* up-regulation after wing injury (Fig S4B and C). To further confirm this unexpected result, we next drove *UAS-TransTimer* with an intronic *drpr* enhancer-*Gal4* (*dee7-Gal4*) that harbors a functional Stat92E-binding site (Fig S4A) and has been shown to up-regulate transcription after brain injury (Doherty et al, 2014). First, we verified that *dee7-Gal4* expression is induced in the brain after antennal injury as described previously (Fig S5A) (Doherty et al, 2014). However, we again failed to observe an increase in the number of GFP^+^ or RFP^+^ puncta 3 d after wing injury in *dee7-Gal4>TransTimer* flies (Fig S5B and C). To extend our investigation to *drpr* levels as a readout of its transcriptional regulation, we measured *drpr* expression in injured wings. *drpr* transcript levels remained unchanged both at 1 and at 3 d post-injury, which is the time of peak Stat92E activity (Fig S5D). Finally, Western blots of Drpr from wing extracts did not show a major difference in abundance relative to the loading control between injured and uninjured states (Fig S5E and F). Thus, *drpr* is not up-regulated in glia by wing injury.

**Figure 3.**
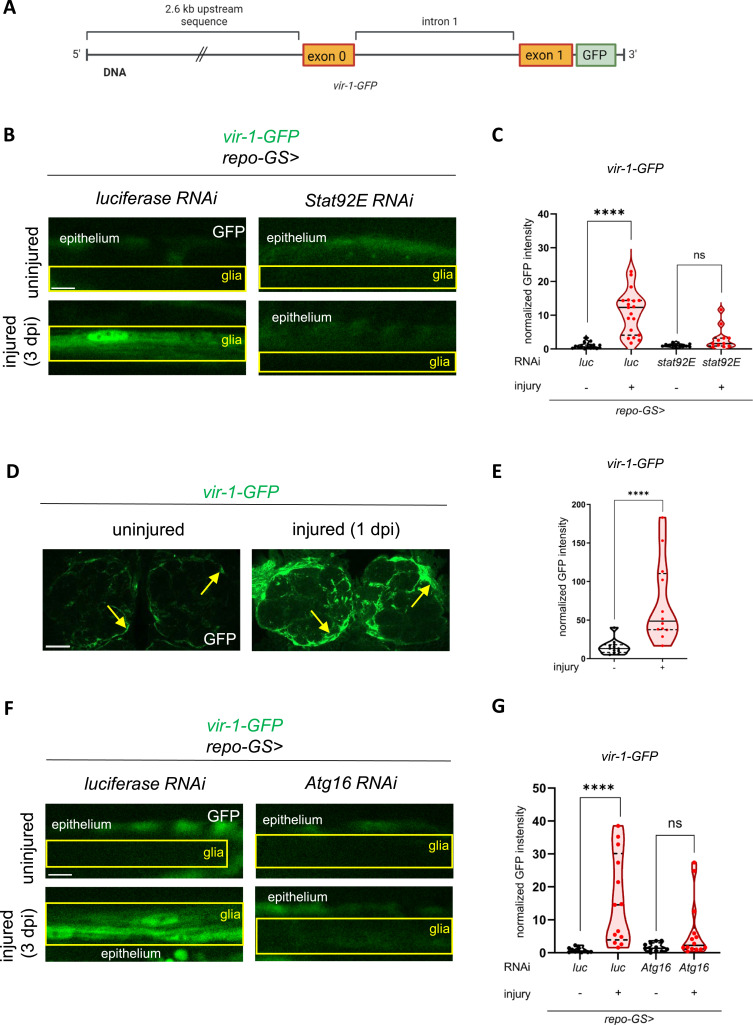
*vir-1* expression is regulated by Stat92E and is controlled by autophagy in glia after wing injury. **(A)** Schematic representation of the *vir-1-GFP* reporter construct: 2.6 kb of *vir-1* upstream sequences drive GFP expression (Dostert et al, 2005). The *vir-1* gene fragment includes the first intron and the first exon. **(B)** Stat92E-dependent activation of *vir-1-GFP* in glia after axonal injury. Single-slice images of wing nerves showing the *vir-1-GFP* signal upon glia-specific *stat92E* knockdown. Scale bar: 5 μm. **(C)** Quantification of *vir-1-GFP* signal shown in (B). An unpaired, two-tailed Mann–Whitney test was used for each pairwise comparison. ****P < 0.0001, ns = 0.0512. n = 16, 19, 12, 11. **(D)** *vir-1* induction in the brain after injury. Single-slice images showing the *vir-1-GFP* signal around the AL, in uninjured animals and 1 d after antennal ablation. Scale bar: 20 μm. **(E)** Quantitative analysis of normalized GFP signal in the brain shown in (D). Data were assessed by using an unpaired, two-tailed Mann–Whitney test. ****P < 0.0001. n = 12, 12. **(F)** *vir-1-GFP* induction upon injury in *Atg16* knockdown flies. Single-slice images of wing nerves showing the *vir-1-GFP* signal upon glial *Atg16* RNAi. Scale bar: 5 μm. **(G)** Quantitative analysis of *vir-1-GFP* signal shown in (F). Unpaired, two-tailed Mann–Whitney test was used for both pairwise comparisons. ****P < 0.0001, ns = 0.1201. n = 11, 13, 11, 14. RNAi expression was induced by RU486 for 5–7 d after eclosion before injury and during the experiment. The boxed area indicates the position of wing nerve glia adjacent to epithelial GFP signal. Truncated violin plots are shown with median and quartiles. Source data are available for this figure.

**Figure S6.**
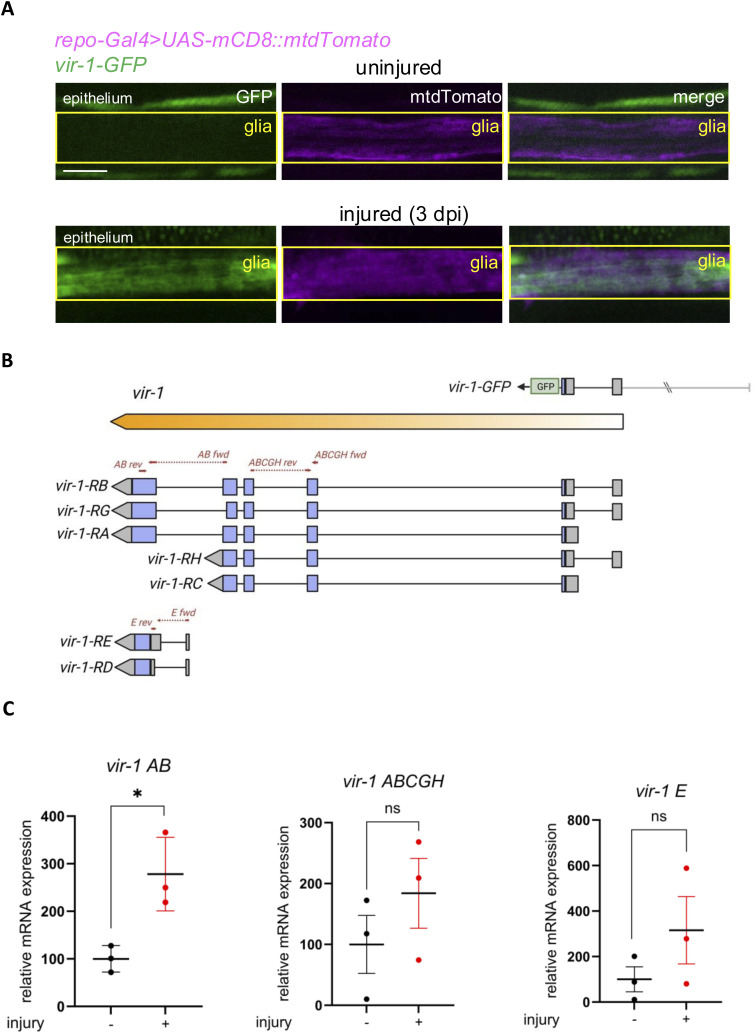
*vir-1-GFP* is expressed in glia after injury. **(A)** Fluorescent microscopic images of uninjured and injured wing nerves 3 dpi. *vir-1-GFP* signal colocalizes with *repo-Gal4*–driven myr::tdTomato^+^ glia after injury. Single optical slices are shown in each case. The arrows show the reporters expressed in glia, whereas the arrowheads denote the epithelium surrounding the veins. The boxed area indicates the position of wing nerve glia adjacent to epithelial GFP signal. Scare bar: 10 μm. **(B)** Schematics of the *vir-1* transcription unit. All transcript variants and the *vir-1*-GFP transgene structure are depicted. **(C)** Primer locations for qPCR in (C) are indicated. **(C)** Transcript levels of different *vir-1* isoforms in wings without injury and 2 d after injury determined by qPCR. *vir-1 AB* indicates *RA* and *RB* transcript isoforms, *vir-1 ABCGH* refers to *RA*, *RB*, *RC*, *RG*, and *RH* isoforms, whereas *vir-1 E* represents the RE isoform. *vir-1* expression levels are normalized to *RpL32* expression. *P = 0.0199, ns (*vir-1 E*) = 0.2433, ns (*vir-1 ABCGH*) = 0.3230. n = 3, respectively.

JAK-STAT signaling underlies many immune pathways including cytokine signaling and defense against viruses (Philips et al, 2022). We thus screened immunity-related gene reporters (Table S2). Among antimicrobial peptides, *Attacin-A* and *Metchnikowin* reporters were only up-regulated in the epithelium after injury. In contrast, *vir-1-GFP* (Dostert et al, 2005) (Fig 3A), a previously characterized transgene, was robustly up-regulated in the wing nerve after injury (Fig 3B and C). GFP expression under the control of the *vir-1* promoter overlapped with myr::tdTomato expressed in glia, which clearly indicates *vir-1* induction in glia (Fig S6A). Stat92E signaling–driven *vir-1* is one of the most strongly induced transcripts after viral infection in *Drosophila* (Dostert et al, 2005). Curiously, *vir-1* is markedly up-regulated in glia specifically 1 d after traumatic brain injury but not later (van Alphen et al, 2022). Brains 1 d post-antennal ablation indeed showed a significant increase in *vir-1* reporter GFP levels in glia in and around the antennal lobe (Fig 3D and E). *vir-1* has seven transcript isoforms. *RE* and *RD* are shorter and encompass only the 3′ half of the longer isoforms (*RA, RB, RG*). Therefore, their transcription initiation is offset relative to these longer variants. We found that after wing injury, *vir-1* isoforms *RA* and *RB* are up-regulated, whereas *RE* is not (Fig S6B and C). This indicates that an injury-responsive enhancer resides in the proximity of the transcriptional start site of the long isoform.

Table S2. Immunity-related gene reporters studied in the wing nerve.

To corroborate that *vir-1* is a Stat92E target in glia, we silenced *Stat92e* acutely in adult glia using *repo-GeneSwitch (repo-GS)* where RNAi expression was induced only in adults to circumvent potential developmental defects (Osterwalder et al, 2001). It also enabled us to reduce *Stat92e* levels in a narrow temporal window starting shortly before injury. *Stat92e* knockdown abrogated *vir-1-GFP* induction in the wing nerve in response to injury (Fig 3B and C). This prompted us to investigate whether autophagy impacts *vir-1* expression, similar to the *10xStat92E enhancer-GFP* reporter. *Atg16* RNAi in glia prevented *vir-1* induction after injury when compared to uninjured wings (Fig 3F and G). Accordingly, autophagy might contribute to glial reactive states at least in part by acting on this important innate immune locus via the regulation of Stat92E.

### Su(var)2-10 depletion enhances Stat92E activation in glia after wing injury

**Figure 4.**
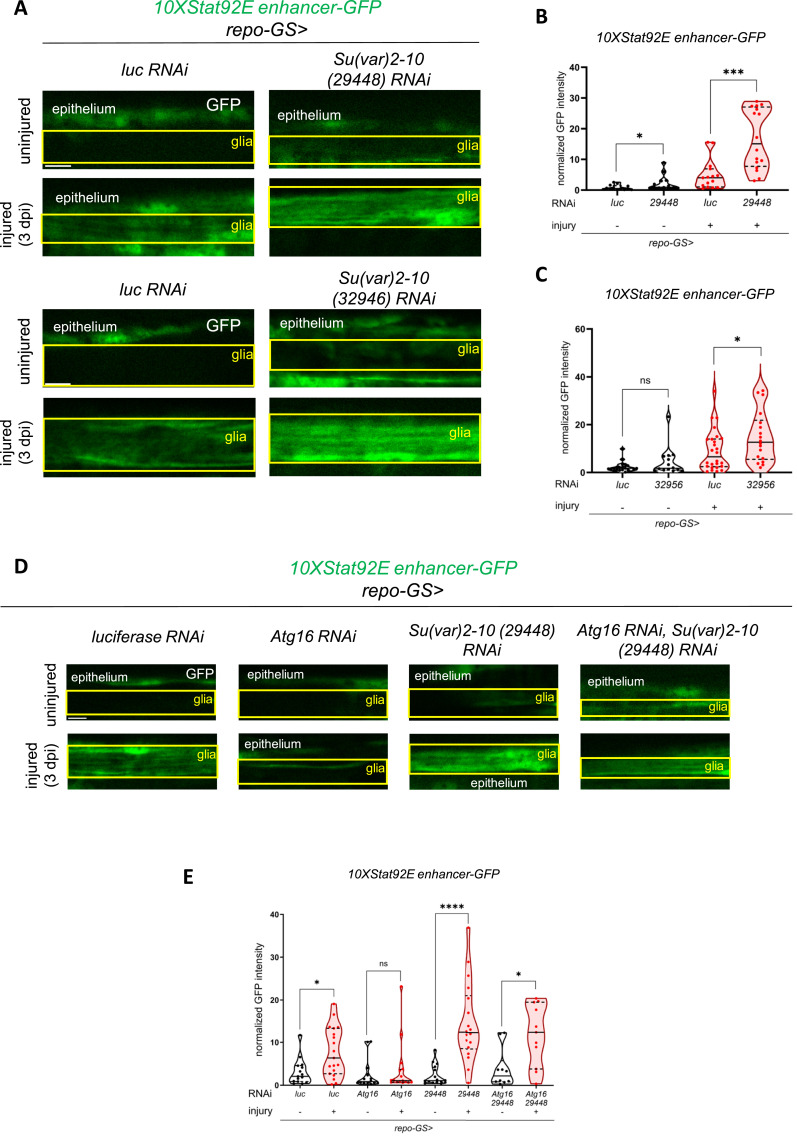
Su(var)2-10 is a Stat92E repressor in glia and mediates the autophagic modulation of Stat92E activity upon injury. **(A)** Single optical slices showing the disinhibition effect of two independent *Su(var)2-10* RNAi constructs on Stat92E activity (*10xStat92E enhancer-GFP*) using the *repo-GS* system in glia. **(B, C)** Quantitative analysis of normalized *10xStat92E enhancer-GFP* signal shown in (A). An unpaired, two-tailed Mann–Whitney test was used for pairwise comparisons. ***P = 0.0002, *P (B) = 0.0120, *P (C) = 0.0482, ns = 0.4742. n (B) = 13, 13, 15, 16. n (C) = 21, 14, 26, 18. **(D)** Epistatic interaction between *Su(var)2-10* and *Atg16*. *Atg16* silencing impairs the activation of Stat92E after injury that is restored by simultaneous *Atg16* RNAi and *Su(var)2-10* knockdown. **(E)** Quantitative analysis of normalized *10xStat92E enhancer-GFP* signal shown in (D). An unpaired two-tailed Mann–Whitney test was used for all comparisons. ****P < 0.0001, *P (*luc*) = 0.0124, *P (*Atg16 + 29,448*) = 0.0127, ns = 0.2939. n = 15, 19, 14, 16, 16, 18, 10, 11. RNAi expression was induced by RU486 feeding for 5–7 d after eclosion, before injury and during the experiment. The boxed area indicates the position of wing nerve glia adjacent to epithelial GFP signal. Scale bar: 5 μm. Truncated violin plots are shown with median and quartiles. Source data are available for this figure.

Next, we screened Stat92E regulators that may be responsible for Stat92E repression in glia. Because the upstream components of the JAK-STAT pathway, Hop and Dome, are not involved in glial injury responses in flies (Doherty et al, 2014), we focused on interactors of Stat92E itself (Myllymäki & Rämet, 2014). A direct repressor of Stat92E is Su(var)2-10/protein inhibitor of activated STAT (PIAS) (Betz et al, 2001), a SUMO ligase whose mammalian orthologs PIAS1-4 SUMOylate and thereby inactivate STAT proteins (Nakagawa & Yokosawa, 2002). We drove two independent *Su(var)2-10* RNAi constructs in adult glia using *repo-GS*. Strikingly, *Su(var)2-10* knockdown relieved Stat92E reporter repression predominantly after injury, but it had a milder effect in uninjured conditions (Fig 4A–C). This raises the possibility that Su(var)2-10 elimination is important for efficient Stat92E-dependent activation after injury.

**Figure S7.**
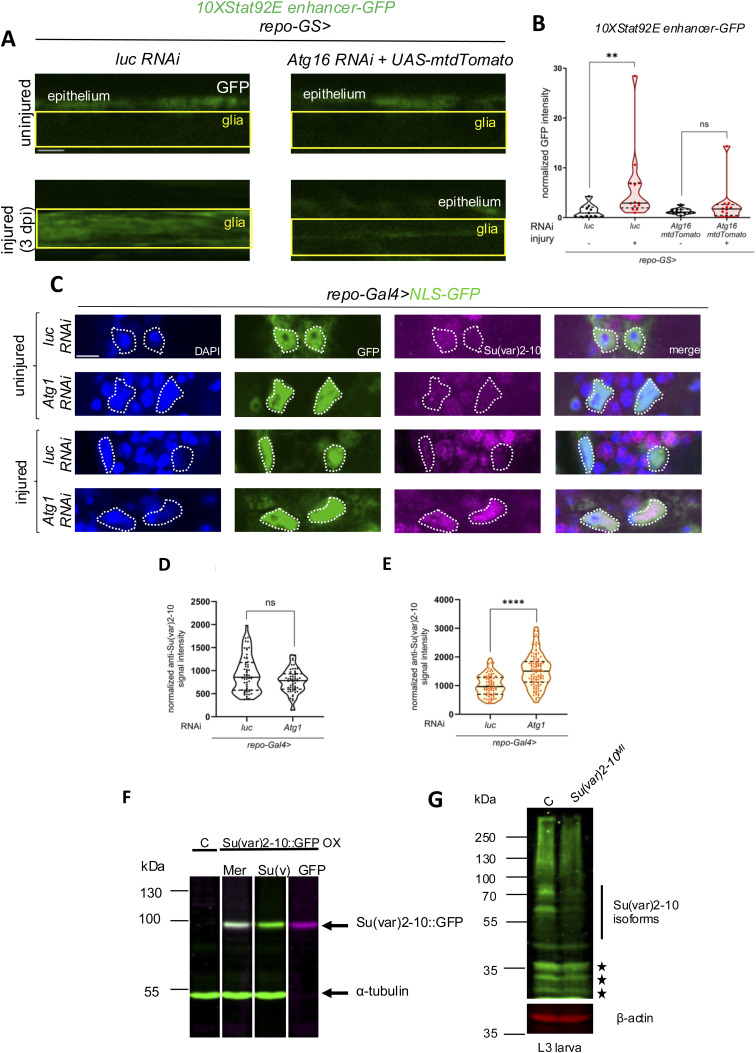
Verification of the Su(var)2-10 antibody and control experiments for Fig 4D. **(A)** Single-slice images of wing nerves showing *10xStat92E enhancer-GFP* signal upon glial RNAi. RNAi expression was induced by RU486 for 5–7 d after eclosion before injury and lasted for the duration of the experiment. *Atg16* RNAi effect is not abrogated by simultaneously introducing a second UAS construct (*UAS-mtdTomato*) arguing against Gal4 unavailability on *Atg16* RNAi and resulting derepression of *10xStat92E enhancer-GFP* in Fig 4D. The boxed area indicates the position of wing nerve glia adjacent to epithelial GFP signal. Scale bar: 5 *µ*m. **(B)** Quantitative analysis of *10xStat92E enhancer-GFP* signal shown in (A). Truncated violin plots are shown with median and quartiles. We used unpaired, two-tailed Mann–Whitney test for the analyses. **P = 0.0033, ns = 0.4491. n = 9, 13, 11, 12. **(C)** Single optical slices showing Su(var)2-10 abundance in brain glia around the antennal lobe in glial *luc* and *Atg1* KD, without injury and 1 d after antennal ablation, respectively. Su(var)2-10 is predominantly nuclear. Glial nuclei labeled by *repo-Gal4>NLS-GFP* are outlined. Note the accumulation of Su(var)2-10 in glial nuclei, and that a fraction of NLS-GFP is also present in the cytoplasm. Scale bar: 5 μm. **(D, E)** Quantitative analysis of Su(var)2-10 nuclear intensity normalized to background signal in single optical slices shown in (C). An unpaired, two-tailed Mann–Whitney test and *t* test were used for statistics for uninjured and injured pairwise comparisons, respectively. ****P < 0.0001, ns, not significant. n (D) = 59, 58. n (E) = 80, 110. **(F)** S2 cell extracts transiently expressing Su(var)2-10-EGFP were immunoblotted for GFP (GFP, magenta) and Su(var)2-10 (Su(v), green) simultaneously. Individual channels and the merged image (Mer) are shown. The signals of the reactive bands completely colocalize indicating recognition of Su(var)2-10 by the Su(var)2-10 antibody. Control extract (C) not expressing Su(var)2-10::GFP is displayed for comparison on the left. Anti-α-tubulin serves as a loading control. **(G)** Homozygous *Su(var)2-10*^*MI03442*^ loss-of-function allele and control (C) *w*^*1118*^ L3-stage larva extracts were immunoblotted for Su(var)2-10 and β-actin. Note the reduced Su(var)2-10 isoform band intensities in the mutant in the >∼45 kD size range. Asterisks indicate bands that are not affected by the MiMIC insertion, most likely nonspecific bands in larva.

Importantly, *Su(var)2-10* depletion enhanced Stat92E activity, whereas the knockdown or abrogation of autophagy genes impaired Stat92E activity in glia. We wondered whether Su(var)2-10 relays the repressive effect of autophagy disruption onto Stat92E reporter activation. We addressed this question via a genetic epistasis experiment. Although knockdown of *Atg16* abrogated *10xStat92E enhancer-GFP* activation, depletion of *Su(var)2-10* in this background restored the normal magnitude of Stat92E response to injury (Fig 4D and E; see also Fig S7A and B for additional controls). This points to Su(var)2-10 as an important mediator of the autophagic regulation of Stat92E-dependent transcription.

### Autophagic degradation of Su(var)2-10 activates Stat92E upon injury

**Figure 5.**
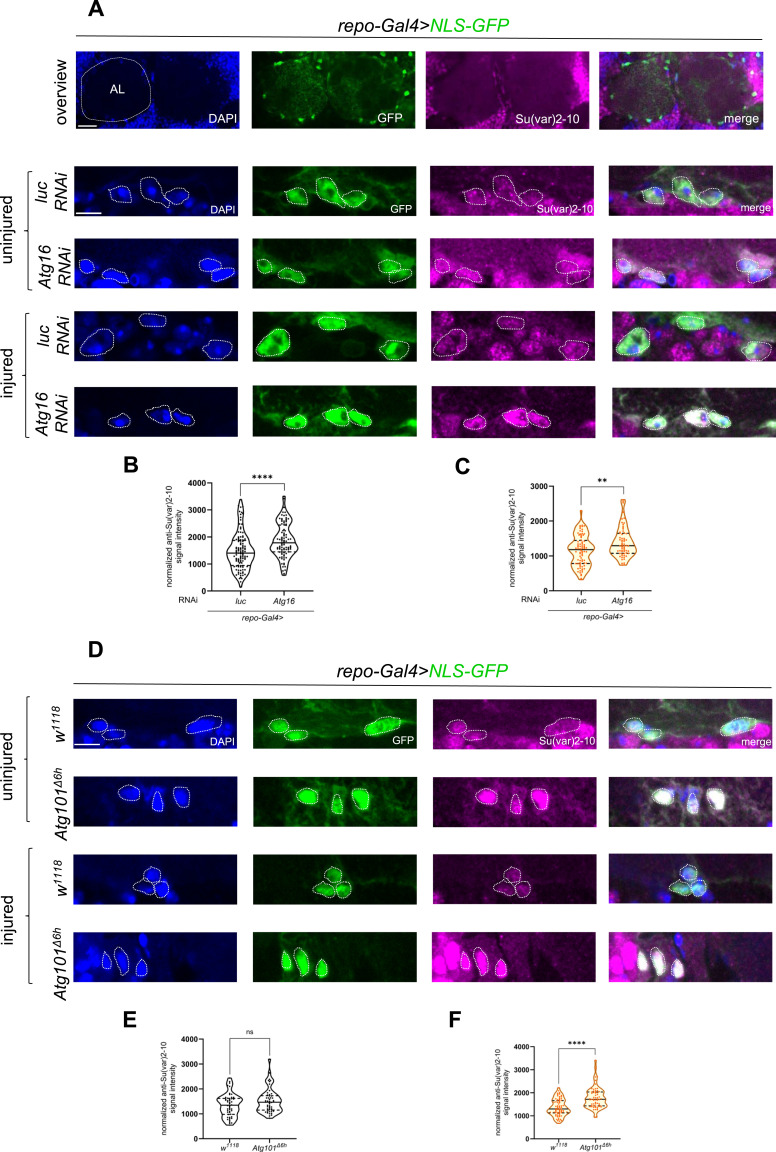
Su(var)2-10 levels are elevated in brain glia upon autophagy inhibition. **(A, D)** Single optical slices showing Su(var)2-10 abundance in brain glia around the antennal lobe (AL) in glial *luc* and *Atg16* KD (A) and *w*^*1118*^ and *Atg101*^*Δ6h*^ mutant (D) background, without injury and 1 d after antennal ablation, respectively. Su(var)2-10 is predominantly nuclear. Glial nuclei are labeled by *repo-Gal4>NLS-GFP*. Arrows denote glial nuclei. Note the accumulation of Su(var)2-10 in glial nuclei, and that a fraction of NLS-GFP is also present in the cytoplasm. Scale bars: 20 and 5 μm. **(B, C)** Quantitative analysis of Su(var)2-10 nuclear intensity normalized to background signal in single optical slices shown in (A). An unpaired, two-tailed Mann–Whitney test was used for statistics. **(B, C)** ****P < 0.0001, **P = 0.0033. n (B) = 109, 90. n (C) = 80, 64. **(E, F)** Quantitative analysis of Su(var)2-10 nuclear intensity normalized to background signal in single optical slices shown in (D). An unpaired, two-tailed Mann–Whitney test was used for statistics. ****P < 0.0001, ns = 0.1289. **(F)** n (E) = 50, 50. n (F) = 70, 60. Truncated violin plots are shown with median and quartiles. Source data are available for this figure.

We further tested the hypothesis that autophagic degradation of Su(var)2-10 leads to Stat92E activation after injury. We depleted *Atg16* in glia and examined Su(var)2-10 protein levels in the adult brain after antennal ablation. Removal of antennae results in axon fragmentation in the antennal lobe that triggers reactivity in surrounding glia (MacDonald et al, 2006). Su(var)2-10 is mainly nuclear, consistent with its transcriptional repressor and chromatin organizer functions (Betz et al, 2001; Hari et al, 2001). We expressed nuclear GFP in glia and inspected the colocalizing Su(var)2-10 pool. Su(var)2-10 levels were elevated in glial nuclei around the antennal lobe upon *Atg16* silencing in both uninjured and injured conditions (Fig 5A–C). This was further validated by examining Su(var)2-10 abundance after antennal ablation in an *Atg101* null mutant in which autophagy initiation is defective (Fig 5D–F). Interestingly, the accumulation of Su(var)2-10 was highly significant in injured mutant brains, whereas the effect was much milder in uninjured brain glia of *Atg101* mutant animals. We wondered whether the differing effect on Su(var)2-10/PIAS accumulation between *Atg16* RNAi and *Atg101* null mutant in uninjured animals is due to their different function in vesicular pathways (Table S1). Therefore, we depleted *Atg1* (encoding the catalytic subunit of the Atg101-containing autophagy initiation complex) in glia, which led to the same lack of Su(var)2-10 accumulation in uninjured conditions as in the *Atg101* null mutant (Fig S7C–E). Strikingly, Su(var)2-10 levels also increased in injured animals with *Atg1* knockdown in glia, similar to *Atg101* mutants. The autophagy initiation complex is highly specific for autophagy, whereas the Atg16-Atg5:Atg12-dependent conjugation of LC3/Atg8a is involved in various vesicle trafficking pathways in addition to autophagy (Nieto-Torres et al, 2021; Figueras-Novoa et al, 2024) (Table S1). This versatile function of the Atg16 complex may perturb baseline Su(var)2-10 levels in uninjured animals as opposed to a specific autophagic dysfunction. We confirmed the specificity of the Su(var)2-10 antibody on Western blots of Su(var)2-10::EGFP ectopically expressed in Schneider 2 R+ (S2R+) *Drosophila* cell culture (Fig S7F) and homozygous *Su(var)2-10*^*MI03442*^ loss-of-function mutant larval extracts (Fig S7G). Taken together, the autophagy-dependent modulation of Su(var)2-10 indicates that it is a selective cargo degraded by autophagy in glia after wing nerve injury.

**Figure S8.**
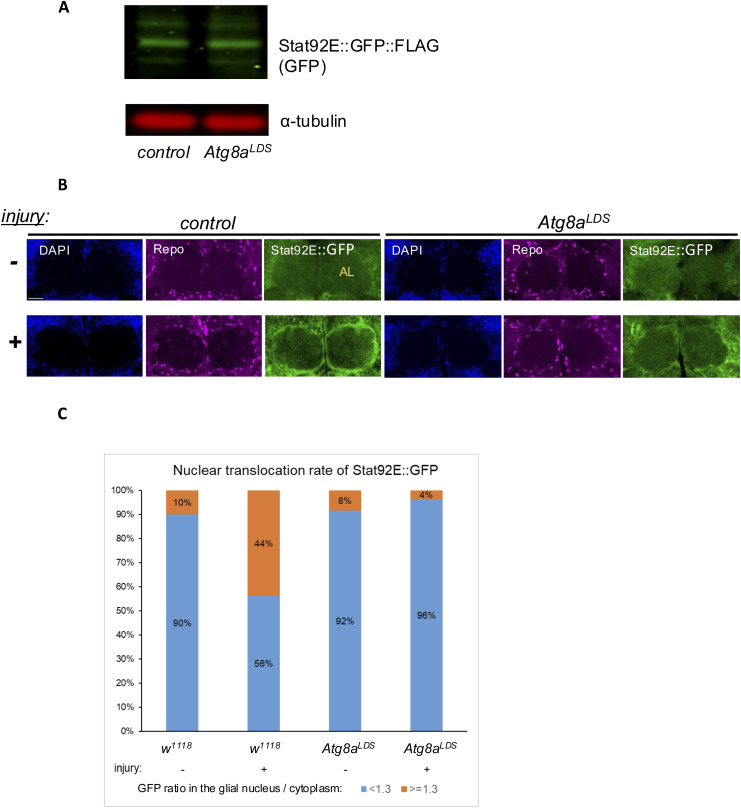
Selective autophagy defect does not change the overall Stat92E::GFP levels in the head but leads to Stat92E nuclear translocation defect. **(A)** Western blot of head lysates of flies expressing STAT92E::GFP under endogenous control in the indicated genetic backgrounds. α-Tubulin serves as a loading control; STAT92E::GFP forms are revealed by anti-GFP. **(B)** STAT92E::GFP signal accumulates in glia surrounding the AL after antennal ablation in control flies but less so in *Atg8a*^*LDS*^ mutants. Scale bar: 20 μm. **(C)** Stacked column chart of the frequencies of the nuclear translocation ratio of Stat92E::GFP above and below 1.3 in *w*^*1118*^ and *Atg8a*^*LDS*^ brain glia after antennal ablation. Stat92E::GFP level is at least 30% higher in glial nuclei compared with their cytoplasm in 44% of all WT brains after injury versus 10% in uninjured conditions. This proportion did not increase in *Atg8a*^*LDS*^ mutants upon injury.

**Figure 6.**
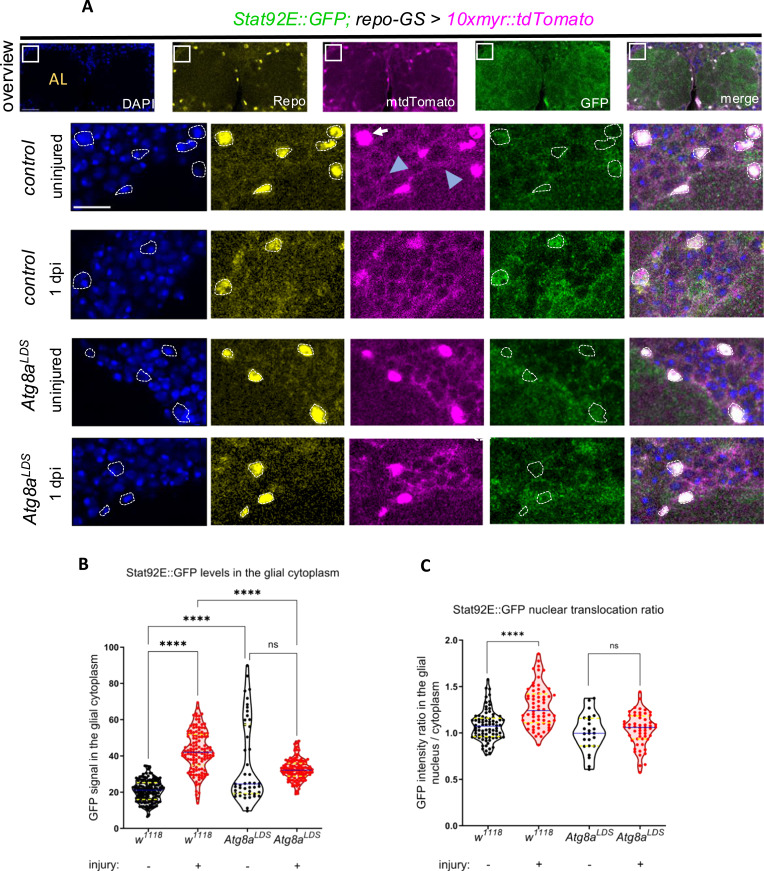
Selective autophagy defect leads to insufficient nuclear translocation of Stat92E. **(A)** Confocal single-slice multichannel images showing the effect of *Atg8a*^*LDS*^ mutation on antennal and maxillary palp ablation-induced nuclear translocation of Stat92E::GFP in ensheathing glia and AL-adjacent cortex glia, outlined by a dashed line. The Stat92E::GFP levels in the glial cytoplasm are also elevated upon injury, which is less pronounced in the mutants. DAPI marks all nuclei, whereas Repo visualizes glial nuclei. Unstained myr::tdTomato marks the glial membranes (blue triangle). Please note that the strong signal coming from Repo-stained nuclei bleeds into the channel of the weak intensity native tdTomato signal denoted by the white arrow. AL, antennal lobe. **(B)** Quantification of Stat92E::GFP levels in the glial cytoplasm. A Kruskal–Wallis test with Dunn’s multiple comparisons test was used for statistical analysis. ****P < 0.0001, ns, not significant, n = 159, 127, 47, 111. **(C)** Quantitative analysis of Stat92E::GFP nuclear translocation in glia. An unpaired, two-tailed Mann–Whitney test and a *t* test were used for statistical analysis. ****P < 0.0001, ns = 0.3969. n = 80, 64, 24, 56. Truncated violin plots are shown with median and quartiles. Scale bars: 20 μm (overview) and 10 μm (magnified images). Source data are available for this figure.

The LIR-binding site mutant *Atg8a*^*LDS*^ could in principle disrupt Stat92E transcriptional reporter up-regulation by lowering Stat92E levels. Using a Stat92E::GFP protein–protein fusion transgene, we found that the levels of the Stat92E protein remained unchanged on Western blots when selective autophagy was impaired (Fig S8A). When we immunostained brains for GFP in Stat92E::GFP animals, we found that Stat92E accumulated in a glial pattern around the antennal lobe (AL) after antennal ablation in WT flies (Fig S8B). This elevation was significantly lower upon block of selective autophagy (Fig 6A and B). We then measured nuclear translocation of Stat92E::GFP in AL-adjacent cortex and ensheathing glia surrounding the antennal lobe. AL degeneration has been shown to induce glial reactivity both in ensheathing and in cortex glia neighboring this region (MacDonald et al, 2013). Although control flies showed a significant nuclear enrichment of Stat92E::GFP after injury, this change did not occur in *Atg8a*^*LDS*^ mutants (Figs 6A and C and S8C). This indicates impaired Stat92E translocation into glial nuclei of *Atg8a*^*LDS*^ mutants after injury.

**Figure 7.**
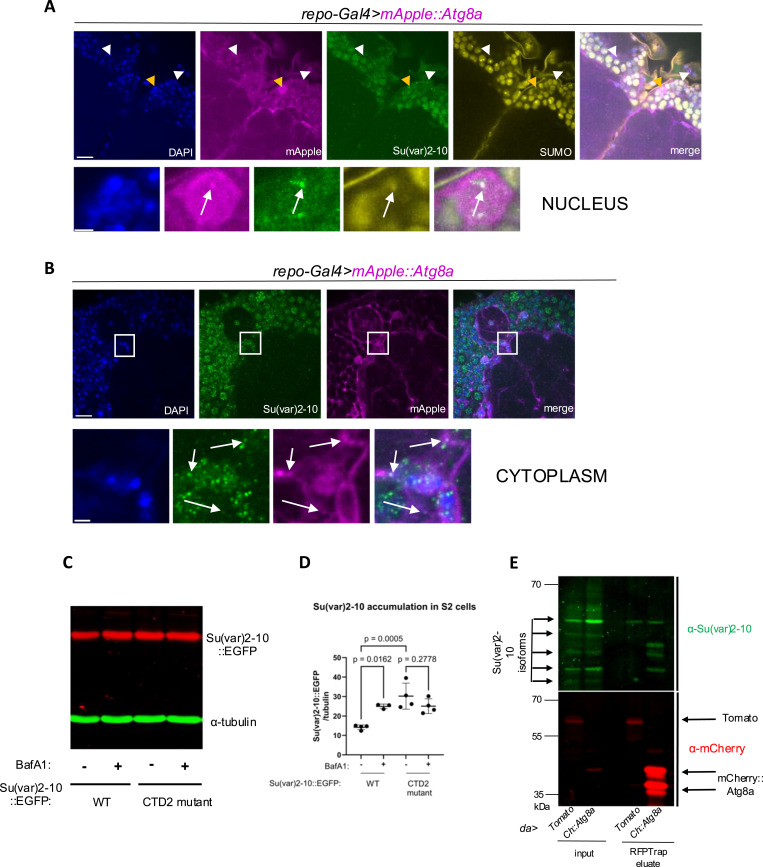
Su(var)2-10 is degraded by autophagy. **(A)** Single optical brain slice around the dorsal antennal lobe area is shown. Magnified images of one typical glial nucleus, denoted by an orange arrowhead, are shown below. Arrowheads point to glial nuclei, whereas arrows denote the exact location of Su(var)2-10-SUMO-Atg8a colocalization in the magnified image. Nuclei are stained with DAPI; magenta indicates native *mApple::Atg8a* signal from expression in glia by *repo-Gal4*, combined with anti-Su(var)2-10 (green) and anti-SUMO (yellow) immunostaining. Scale bar for brain images: 10 μm; scale bar for nucleus images: 2 μm. **(B)** Confocal single-slice multichannel images show Su(var)2-10-Atg8a colocalization in the cytoplasm of glial cells. Magnified images of the areas outlined by the rectangles can be seen below. Arrows point to the exact location of the colocalization. Nuclei are stained with DAPI; *mApple::Atg8a* expression is driven in glia by *repo-Gal4*, and its signal was amplified by anti-mCherry staining, jointly with anti-Su(var)2-10 immunostaining. Scale bar for brain images: 10 μm; scale bar for nucleus images: 2 μm. **(C)** S2 cells transiently expressing WT and autoSUMOylation-defective CTD2 mutant Su(var)2-10::EGFP were treated with bafilomycin A1 (BafA1) or the solvent DMSO for 4 h, and total Su(var)2-10 levels were assessed by anti-GFP Western blot. α-Tubulin serves as a loading control. **(D)** Quantification of Su(var)2-10 abundance normalized to α-tubulin as in (C). Shown are the mean and SD; one-way ANOVA with the Holm–Šídák test for multiple comparisons correction was used for statistics. n = 4, 3, 4, 4. **(E)** Co-immunoprecipitation of Su(var)2-10 with mCherry::Atg8a. Ubiquitous *daughterless* (*da*)-*Gal4*–driven myr::tdTomato (Tomato) or mCherry::Atg8a (Ch::Atg8a) whole adult fly extracts were precipitated with RFP-Trap beads. myr::tdTomato serves as a negative control. Eluates are shown on the right, and crude extract inputs (10 μg) on the left. The two major bands in mCherry::Atg8a correspond to the lipidated and unlipidated forms. Western blots were probed by anti-mCherry and anti-Su(var)2-10. Source data are available for this figure.

We then investigated how autophagy may regulate Su(var)2-10 levels. Su(var)2-10, similar to previous studies (Hari et al, 2001), was present in small foci in the nucleus and to a lesser extent in the cytoplasm in brains (Fig 7A). We noticed that nuclear Su(var)2-10 often colocalizes with glia-expressed Atg8a in a punctate pattern (Fig 7A), raising the possibility that these proteins interact with each other. Although we did not find a predicted consensus LIR motif in Su(var)2-10, its interaction with Atg8a might be mediated by other motifs (Gohel et al, 2020) or via their binding partners. Su(var)2-10 is known to autoSUMOylate itself (Ninova et al, 2020; Bence et al, 2024). Indeed, SUMO colocalized with structures that also contain Su(var)2-10 and Atg8a (Fig 7A). SUMO-modified proteins accumulate in concert with PIAS activity in phase-separated foci, which are also referred to as SUMO glue (Gutierrez-Morton & Wang, 2024). We found that about 50% of glial cells contain triple-colocalizing Su(var)2-10-SUMO-Atg8a puncta. Su(var)2-10 degradation must occur in the cytoplasm if it is an autophagy cargo (Gohel et al, 2020). Indeed, cytoplasmic structures double positive for Su(var)2-10 and Atg8a were readily detected in glia surrounding the AL (Fig 7B).

To gain more insight into the degradation mechanism, we next tested Su(var)2-10 degradation in a heterologous system by transfecting cultured *Drosophila* S2R+ cells with *Su(var)2-10::EGFP* in the presence or absence of bafilomycin A1 (BafA1) that inhibits both autophagosome fusion and lysosomal acidification. Su(var)2-10 levels increased upon BafA1 treatment, which supports the lysosomal degradation of this protein (Fig 7C and D). We then wondered whether autoSUMOylation of Su(var)2-10 is the lysosomal degradation signal. The C-terminal domain (CTD) of Su(var)2-10 interacts with SUMO. Mutating two residues in the CTD2 region disrupts SUMO binding and the autoSUMOylation of Su(var)2-10 (Bence et al, 2024). This leads to a decreased nuclear-versus-cytoplasmic Su(var)2-10 ratio in vivo. Using this construct, we found that baseline levels of the CTD2 mutant form are increased relative to the WT protein, hinting to a degradation defect (Fig 7C and D). Strikingly, CTD2 mutant protein levels were insensitive to BafA1 treatment, which strongly suggests that SUMOylation-deficient Su(var)2-10 cannot undergo lysosomal degradation.

Finally, we aimed to reveal whether Su(var)2-10 physically interacts with Atg8a in vivo, because Atg8a serves as an interaction platform for autophagy cargos and cargo receptors. We indeed detected a specific interaction between certain Su(var)2-10 isoforms and tagged Atg8a by immunoprecipitation from whole fly extracts (Fig 7E). Collectively, our experiments indicate that interaction of Su(var)2-10 with Atg8a in the nucleus and in the cytoplasm can trigger its autophagic breakdown, which involves Su(var)2-10’s SUMOylation.

## Discussion

Here, we have uncovered that down-regulation of a direct repressor of the Stat92E transcription factor, and thus Stat92E activation and nuclear translocation, is mediated by autophagy. This, synergistically with injury-induced Stat92E phosphorylation, may ensure proper Stat92E activity in glia after nerve injury to promote glial reactivity (Fig 8).

The significance of the vital role of autophagy in glial biology is just beginning to unfold (Sung & Jimenez-Sanchez, 2020; Qin et al, 2021; Nagayach & Wang, 2023). Neuroprotective microglia in the aging brain show elevated levels of autophagy (Berglund et al, 2024). Autophagy limits neuroinflammation, for example, by degrading the inflammasome component NLRP3 in microglia to prevent cytokine release and proinflammatory responses (Houtman et al, 2019). We find that in addition to these functions, autophagy can also positively regulate glial reactivity by impacting a major immune signaling pathway. Selective autophagic elimination of Su(var)2-10 and autophagy-dependent nuclear translocation of Stat92E provide an alternative to canonical JAK-dependent Stat92E regulation that is apparently not employed in glial injury signaling (Doherty et al, 2014). How injury regulates selective autophagy to remove Su(var)2-10 is an open question. Bulk autophagy is not elevated in glia after wing injury; however, specific degradation signals such as ubiquitylation of selected cargos can still induce their degradation by selective autophagy (Kraft et al, 2010; Goodall et al, 2022). We suspect that SUMO may fulfill a similar dynamic role in labeling cargo for elimination and this modification may be promoted by injury in the case of Su(var)2-10.

Depletion of members of the autophagy-specific initiation complex, *Atg101* and *Atg1*, leads to Su(var)2-10 accumulation in glia only after brain injury. The *10xStat92E enhancer-GFP* reporter data suggest that if Su(var)2-10 is eliminated from glia, a significant increase in Stat92E activity is only seen when flies are simultaneously injured. Su(var)2-10 removal alone is probably insufficient to trigger Stat92E activation because Stat92E activation still depends on injury-induced tyrosine phosphorylation (Ekas et al, 2010; Doherty et al, 2014) for its proper function in reactive glia. Therefore, we believe that Stat92E operates as a coincidence detector: integrating signals from injury-induced autophagic removal of Su(var)2-10 and activating phosphorylation for full activation. Drpr signaling through Rac1 may induce Stat92E activity via Stat92E phosphorylation (Doherty et al, 2014), and Drpr activation could also lead to enhanced SUMOylation of Su(var)2-10 to stimulate its degradation.

The idea of selective autophagic elimination of Su(var)2-10 is supported by its lysosomal degradation, which does not happen in case of an autoSUMOylation-defective mutant form. SUMOylation regulates many critical developmental and cellular processes and enables quick responses to environmental and homeostatic changes. Of note, autophagy also contributes to the elimination of SUMOylated Ataxin-3 (Hwang & Lee, 2017). Ubiquitylation of SUMOylated proteins by SUMO-targeted ubiquitin ligases (STUbLs) can indeed lead to subsequent degradation (Abed et al, 2018; Han et al, 2023).

STAT proteins can also be subject to elimination by autophagy under pathological conditions. A bat coronavirus effector, the E protein, compromises host immune reactions by enhancing autophagic degradation of STAT2 via the OPTN and NBR1 SARs (Huang et al, 2025). Su(var)2-10 being a predominantly nuclear protein and autophagy assumed to be cytoplasmic, we need an explanation for autophagic breakdown of Su(var)2-10. Nuclear autophagy is a bourgeoning concept where nuclear structural constituents such as lamin B1 (Dou et al, 2015) and transcriptional and chromatin regulators such as SIRT1 and WSTF (Xu et al, 2020; Wang et al, 2025) are recognized by direct LC3 binding and transported out of the nucleus for cytoplasmic degradation via autophagy. WSTF elimination triggers chronic inflammation. Interestingly, SUMOylation of lamin B1 enhances its autophagic degradation upon spinal cord injury (Fan et al, 2024).

We find that Su(var)2-10 colocalizes with Atg8a both in the cytoplasm and in the nucleus of brain glia. Nuclear Su(var)2-10 also colocalizes with both SUMO and Atg8a. LC3/Atg8a nuclear pools are important for autophagy activation (Huang et al, 2015; Jacomin et al, 2020). We do not expect a full overlap between Su(var)2-10 and Atg8a labeling for a number of reasons. First, Su(var)2-10 has many different roles that may not be regulated by autophagy. Second, Atg8a-positive autophagosomes in the cytoplasm deliver not only individual proteins such as Su(var)2-10 for degradation but also many other cellular components. Third, nuclear Atg8a is implicated in the removal of the Sequoia transcriptional repressor from autophagy genes that is unlikely to involve Su(var)2-10 (Jacomin et al, 2020). Given that vesicles are excluded from the nucleus, Su(var)2-10-SUMO-Atg8a triple-positive structures are likely LLPS multimolecular aggregates. SUMOylation regulates stress granule assembly (Marmor-Kollet et al, 2020) and other types of phase separation (Lascorz et al, 2022), and the Su(var)2-10 ortholog PIAS1 is predicted to undergo LLPS (Pu et al, 2022 *Preprint*).

**Figure S9.**
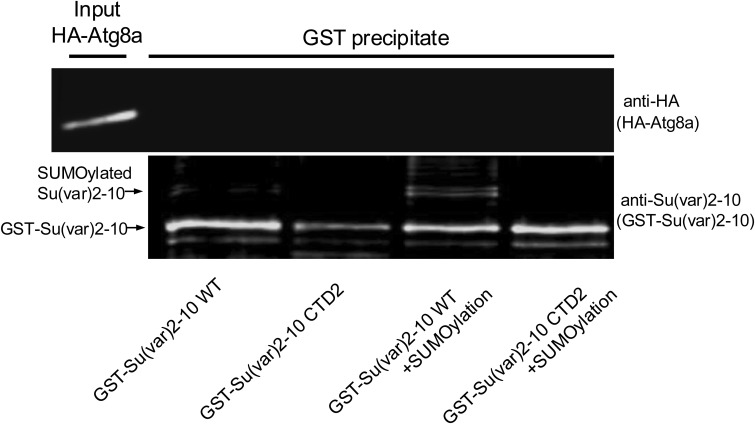
GST pull-down experiments fail to show Atg8a interaction with Su(var)2-10. Purified GST-Su(var)2-10 was in vitro–SUMOylated. Unmodified and SUMOylated WT and SUMOylation-defective CTD2 variant Su(var)2-10 were incubated with in vitro–transcribed/translated HA-Atg8a, and interactions were tested by GST pull-down and anti-HA Western blotting.

The lack of a LIR motif suggests that Su(var)2-10 does not interact directly with Atg8a: it probably requires further labels/interactors that mediate its elimination by autophagy. In support for this hypothesis, we failed to find a physical interaction in vitro between Su(var)2-10 and Atg8a in GST pull-down assays, irrespective of the SUMOylation status of Su(var)2-10 (Fig S9). Nonetheless, the Stat92E activation defects in the *Atg8a*^*LDS*^ mutant, the SUMOylation-dependent lysosomal degradation of Su(var)2-10, and its colocalization and biochemical interaction with Atg8a all support the selective autophagic clearance of Su(var)2-10.

The sensitivity of reporters measuring Stat92E -dependent transactivation is crucial for the investigation of Stat92E regulation. The most sensitive Stat reporter described in *Drosophila*, *10xStat92E enhancer*-*dGFP*, reports transcription dynamics in an hours timescale. However, we were unable to detect signal from this reporter in the wing nerve after injury, likely because of its rapid protein turnover combined with low level of transcription. Therefore, we resorted to using stable GFP (*10xStat92E enhancer*-*GFP*), which still reports dynamic up- and down-regulation of Stat92E activity during the days after injury.

We find that with a similar stable GFP-encoding reporter the alleged antiviral factor *vir-1* (Dostert et al, 2005) is strongly induced by Stat92E in glia after axon injury, both in the wing nerve and in the brain. *vir-1* (CG5453) expression has been previously documented in a subset of glia where it is a target of Gcm, a master regulator of glial cell fate (Freeman et al, 2003). Importantly, it is also used as a longitudinal glial cell marker in the embryo (Ahn et al, 2014). A recent study showed strong up-regulation of *vir-1* in glia after closed head traumatic brain injury, similar to antimicrobial peptides (van Alphen et al, 2022). Its expression may be differentially regulated among glial classes because *vir-1* expression defines a distinct subset of fly macrophages (Cattenoz et al, 2020). Indeed, *vir-1-GFP* is induced in ensheathing glia and possibly astrocytes in the brain after injury (Fig 3E) based on the morphology of GFP^+^ cells. Vir-1 localizes to the extracellular space in the foregut (Zhu et al, 2024), but its roles are not known.

Glial reactivity in *Drosophila* likely relies largely on Stat92E signaling, because in the CNS, Stat92E induces *drpr* and *matrix metalloproteinase 1* (Mmp1) expression that promotes phagocytosis and access to cell debris via ECM degradation (Doherty et al, 2014; Purice et al, 2017). Furthermore, *stat92e*-deficient glia do not even extend processes toward injured axons (Doherty et al, 2014). Correspondingly, phosphorylated STAT3 is a marker for reactive astrocytes in mice (Escartin et al, 2021). Illustrating the complex functionality of this pathway in mammals, STAT3-dependent transcription has a protective role during traumatic brain injury, motor neuron injury, spinal cord injury, and neonatal white matter injury, whereas it contributes to degeneration in AD models (Escartin et al, 2021). Accordingly, glial Stat92E activation is also observed in an amyloid-beta–expressing *Drosophila* model (Ray et al, 2017). We find that selective autophagy promotes Stat92E signaling, and thereby, it could assist functional recovery after injury. In line with this, autophagy deficiency in microglia predisposes to increased neurodegeneration and demyelination in a multiple sclerosis (MS) model (Berglund et al, 2024). Based on our findings, we expect that the benefits of autophagy stimulation in the nervous system will partly manifest through STAT transcription factor–driven glial reactivity.

## Materials and Methods

### *Drosophila* stocks and maintenance

Flies were kept at 25°C on a medium containing cornmeal, yeast, agar, and dextrose with Nipagin as a preservative in reusable glass vials and bottles. For our experiments, we injured male *Drosophila melanogaster* that were 2–7 d old after eclosion. During our experiments, we favored male flies to avoid technical inconvenience related to egg laying and to prevent larvae from making the medium too liquid and sticky. The animals were anesthetized by carbon dioxide. Progeny of *repo-GS* crossed with RNAi lines were raised on normal food during development, and then, males were kept for 5–7 d on food supplemented with 25 μg/ml RU486/mifepristone (Acros Organics) before axotomy and thereafter during the course of the experiment. Injury was carried out on 7- to 12-d-old flies. The supplemented medium used for experiments using *vir-1-GFP* contained 125 μg/ml RU486.

The following stocks were ordered from the Bloomington *Drosophila* Center: *repo-Gal4 (7415)*, *w*^*1118*^, *daughterless-Gal4 (95282)*, *vasa-Cas9* (*51323*), *TRE-EGFP (59010)*, *firefly luciferase*^*JF01355*^, *Atg8a*^*TKO.GS04635*^ *(80828)*, *Su(var)2-10*^*JF03384*^ *(29448)*, *Su(var)2-10*^*HMS00750*^ *(32956)*, *Atg1*^*GL00047*^ *(35177)*, *UASp-mCherry::Atg8a (37750)*, *UASp-GFP-mCherry-Atg8a (37749)*, *UAS-TransTimer (93411)*, *drpr*^*MI07659*^ *(843909)*, *UAS-2xEYFP (60291)*, vas-int, w[*], *hs-Cre (60299)*, *pP{lox(Trojan-GAL4)x3} (60310)* (Diao et al, 2015), *Su(var)2-10*^*MI03442*^ *(37190)*, *Stat92E*^*GL00437*^ *(35600)*, *10XStat92E-ΕGFP (26197)*, *10XStat92E-dGFP (26199, 26200)*, *Stat92E::GFP.FLAG (38670)*, *UAS-NLS::GFP (4775)*, *Atg16*^*MI00187*^ *(30656)*, and *10XUAS-IVS-myr::tdTomato (32221)*.

The following stocks were provided by the Vienna *Drosophila* Resource Center: *Rubicon*^*KK108247*^, *Atg13*^*KK100340*^, *Atg16*^*KK10232*^, and *ref(2)P*^*KK105338*^. The *Atg5*^*5cc5*^ (Kim et al, 2016), *Atg8a*^*LDS*^ (Rahman et al, 2022), *Atg5*^*5cc5*^*; Atg5::3xHA* ^24^ stocks were formerly created and published. *Atg101*^*Δ6h*^ was contributed by Wanzhong Ge (Institute of Genetics, Zhejiang University School of Medicine, Hangzhou, China) (Guo et al, 2019). The following stocks received as gifts were much appreciated: *repo-GeneSwitch* was kindly shared by Véronique Monnier (Université de Paris, BFA Unit of Functional and Adaptative Biology, UMR 8251, France), *OK371-Gal4*, *UAS-mCD8-GFP* was a kind gift from H. Aberle (Institute of Functional Cell Morphology, Heinrich- Heine-University, Düsseldorf). *dee7-Gal4* (*drpr* enhancer-*Gal4*) was kindly contributed by Marc Freeman (Vollum Institute, Oregon Health and Science University, USA), the *Atg8a*^*LDS*^ stock by Ioannis Nezis (University of Warwick, UK), and *vir-1-GFP.2.6 (vir-1-GFP)* by Jean-Luc Imler (IBMC, University of Strasbourg, France). Knockdown efficiency of *Atg* gene RNAi lines was validated previously (Szabó et al, 2023). We have published *nSyb-lexA.DBD::QF.AD*, *lexAop2-CD4::tdTomato* (Szabó et al, 2023).

The *Atg8a*^*null*^ frameshift mutant was generated by CRISPR/Cas9 editing. To generate this null allele (*Atg8a*^*Δ12*^), we used the *Atg8a*^*TKO.GS04635*^ gRNA line and crossed it with *vasa > Cas9* transgenic flies. We screened candidate mutant lines by PCR and Sanger sequencing and identified the *Atg8a*^*Δ12*^ allele that carries an 8-bp deletion based on sequencing data (Fig S2A), which causes frameshift in all isoforms of the Atg8a gene.

The 2xmApple::Atg8a construct was cloned into the pACU vector (#58373; Addgene). mApple sequences were amplified from the pCDH-TMEM106b-mApple vector (#179385; Addgene). The PCR products that were used for the construct assembly with the NEBuilder HiFi DNA Assembly Master Mix (New England Biolabs) are listed in Table S3. We validated the constructs by Sanger sequencing, and we were able to detect mApple fluorescence in induced transfected S2 cells cotransfected with pMT-Gal4. The transgenic *Drosophila* stock was established by the *Drosophila* Injection Service in the Biological Research Center, Szeged (Hungary). The transgene was inserted into the *attP40* (2L) landing site.

Table S3. List of primers used in this study.

A MiMIC transgene (*MI07659*) located in intron 6 of *drpr* was converted into a *drpr-Gal4* fusion gene using a *Gal4* donor so that Gal4 is produced instead of most of the *drpr* coding sequence. We followed the protocol described in Diao et al (2015) by crossing in recombinase and donor transgenes. A donor cassette containing a splice acceptor (*SA*), the *Gal4* to be encoded in the correct frame, and finally a stop codon and polyadenylation site between two *attB* sites was mobilized and circularized by heat shock–inducible Cre. Integration into *MI07659* was achieved by vas-dΦC31. Candidate progeny were screened for *drpr* expression by crossing to *UAS-2xEYFP*. Integration into *drpr* was validated by PCR. *drpr-Gal4* is expressed in glia in the CNS and PNS in addition to other tissues.

We used *repo-GS* in all cases where *repo-Gal4* expression did not give any viable progeny or when the gene targeted by RNAi was known to play a role in crucial developmental processes.

### Rearing axenic stocks for qRT–PCR

We placed ∼100 animals from the used strains into a cylinder containing black egg-laying agar coated with yeast for one night. Embryos were dechorionated in 4–5% hypochlorite on a filter for 1 min followed by washing with flowing distilled water. Finally, we removed the filter fabric, placed it on a paper towel, and transferred the eggs into new vials containing a nutrient medium mixed with antibiotics already described (Tusco et al, 2017). Autoclaved cornmeal–yeast–agar–dextrose–Nipagin medium was mixed with 0.25 mg/ml ampicillin, tetracycline, streptomycin, and 1 mg/ml kanamycin (Tusco et al, 2017). Flies were kept for at least three generations on antibiotics. The concentration of the antibiotics was reduced to half 20 d before sample collection to increase the amount of progeny. The food cocktail preparation and fly transfer took place in a laminar box using only autoclaved and sterile tools.

### Wing injury

In all experiments we carried out in *Drosophila*, male flies were used. In the wing injury assay, we used the axotomy model developed by Fang et al (2012) and Neukomm et al (2014). In accordance with the model (S1A), we removed half of the wing of flies from the distal end with a straight cut across using microsurgical scissors (Fine Science Tools). For each individual, we only cut one wing, leaving the other intact as uninjured control. The injured animals were then kept in vials at 25°C. Three days after the injury (3 dpi), a strong *10xStat92E enhancer-GFP* signal was already obvious (Fig S1D and E).

### Antennal ablation

The antennae were ablated 1 d before brain dissection by pulling on with a forceps (MacDonald et al, 2006), and the injured flies were kept in vials at 25°C.

### Immunostaining of adult brains

Immunostaining of adult brains was performed as described with some modifications (Wu & Luo, 2006). Adult brains were dissected in ice-cold PBS and placed immediately in 4% PFA in PBS with 0.3% Triton X-100 (PBT) on ice. Brains were fixed for 1 h at 25°C. After two quick rinses with PBT, brains were washed three times 20 min each. After blocking in 5% FBS in PBT for 1 h at RT, brains were incubated with the primary antibody (mouse anti-Su(var)2-10 (Andreev et al, 2022), 1:100; rabbit anti-GFP, 1:1,000, A-11122; Thermo Fisher Scientific; mouse anti-Repo, 8D12, 1:25; rabbit anti-SUMO (Gonzalez et al, 2014), 1:200; rabbit anti-mCherry, NBP2-25157, 1:500; Novus Biologicals) for 3 d at 4°C in 5% FBS in PBT. The SUMO antibody was gently provided by Giacomo Cavalli (Institute of Human Genetics [IGH], Montpellier, France) and the Su(var)2-10 antibody by Julius Brennecke (IMBA, Vienna, Austria). Washes were done as before, and brains were incubated with the fluorescently labeled secondary antibody (goat anti-rabbit Alexa Fluor 488, A-11034, goat anti-mouse Alexa Fluor 568, A-11031, from Thermo Fisher Scientific, all 1:1,000 diluted) in 5% FBS in PBT for 2 d at 4°C in darkness. The secondary antibody was removed by two quick rinses, and DAPI (1:15,000) was put into the following first wash with PBT. Brains were washed three times 20 min each. After the washes, the brains were mounted in Vectashield (H-1000-10; Vector Laboratories), and the samples were kept in darkness at 4°C until imaging.

### Imaging

For imaging reporter signals in the wing, we focused on a specific region inside the L1 vein proximal to the injury site, a curvature from the wing margin toward the inner area of the wing, containing axons and glial cells (Fig S1A). We analyzed the samples at RT immediately after dissection and mounting into Halocarbon Oil 27 (H8773; Sigma-Aldrich), with an Axio Imager.M2 structured illumination microscope (Zeiss) and the ORCA Flash4.0LT CMOS camera (Hamamatsu) using a Zeiss Plan-Apochromat 63x/1.40 NA objective. Optical slices were created with a Zeiss ApoTome.2 device. The illumination was provided by the CoolLED pE-4000 system. We captured the images using the Zeiss ZEN program.

For measuring the Su(var)2-10 signal in Su(var)2-10 and NLS-GFP co-immunostained brains, the Visitron VisiScope Spinning Disk Confocal Microscope (Visitron Systems GmbH) was used. The optical slices were created with an Olympus LUCPlanFl 40x NA 0.60 objective for quantification, whereas magnified images shown were captured with an Olympus PlanApo 60x NA 1.42 (oil) objective. Detection was based on an Andor Zyla 4.2 PLUS camera.

The images for Fig 6 were created with the Leica Stellaris 8 confocal microscope with Diode 405 and white light laser (WLL) excitation. The following lasers, excitations, and emission spectra were used for the four channels: Diode 405 nm, 431–508 nm for DAPI; WLL 499 nm, 508–568 nm for GFP; WLL 579 nm, 588–642 nm for 10xmyr::tdTomato; WLL 663 nm, 675–800 nm for Repo. The optical slices were created with HC PL FLUOTAR 40x/0.80 DRY objective for quantification, whereas magnified images shown were captured with HC PL APO CS2 63x/1.40 OIL objective with a 1.00-mm pinhole.

For the three- and four-channel fluorescence images in Fig 7, 405-nm excitation with 405/488/561 triband emission filter, 488-nm excitation with 525/50 emission filter, and 561-nm excitation with 605/70 emission filter were used with a triple-band 405/488/561 dichroic mirror. For the four-channel fluorescence images, 405-nm excitation with 405/488/561 triband emission filter, 488-nm excitation with 525/50 emission filter, 561-nm excitation with 605/70 emission filter, and 640-nm excitation with 700/75 emission filter were used with a quad band 405/488/561/640 dichroic mirror. Channels were acquired in sequential mode, after the performed Z sectioning. We used a disk with 50-μm pinholes.

For *vir-1-GFP* signal detection in brain, we used an LSM800 (Zeiss) inverted laser scanning confocal microscope. The brains were imaged at RT with a Zeiss Plan-Apochromat 40x/1.3 NA oil immersion objective with a Z-step of 2.00 μm. The same microscope settings (illumination, exposure time, laser intensity) were used for all conditions and genotypes throughout one experiment.

### RNA purification and qRT–PCR

For RNA isolation, we collected around 150-200 wings from axenic flies (*vir-1*) or flies raised on normal food (*drpr*) per sample using only autoclaved tools with autoclaved wiping papers placed on the fly sorting pad previously washed with 70% ethanol. The wings were swept in TRI Reagent (Zymo Research)–containing microcentrifuge tubes, using a funnel. We homogenized the samples directly after putting them into TRI Reagent. Total RNA was purified with the Direct-zol RNA Microprep (Zymo Research). DNase I digestion was also applied. 76–80 ng total RNA was reverse-transcribed in 20 μl reaction volume with RevertAid First Strand cDNA Synthesis Kit (Thermo Fisher Scientific) with random hexamer primers. qRT–PCR was performed in 20 μl reactions in technical triplicates using PerfeCTa SYBR Green FastMix (Quantabio) with 0.5 μl cDNA and cycled on a Rotor-Gene Q qPCR machine (QIAGEN) running Rotor-Gene software 2.3.1.49 (QIAGEN), with the following program: 95°C, 3 min; 45 cycles of 95°C, 20 s, 60°C, 20 s, and 72°C, 20 s, followed by melting curve analysis. The ΔΔCt method was employed to normalize the data, with *Ribosomal protein L32* (*RpL32*, also known as *rp49*) serving as the internal control. All primers were designed with Primer-BLAST (https://www.ncbi.nlm.nih.gov/tools/primer-blast) with amplicon length set to 70–150 bp. The used melting temperature was 60°C. One primer spanned an exon–exon junction in all cases. Primers used for qRT–PCR are listed in Table S3. We normalized the qRT-PCR datasets to RpL32. The ΔCt-derived expression values were adjusted by a common scaling factor to ensure that the average of control values equaled 1 or 100.

### *Drosophila* S2R+ cell culture

*Drosophila* S2R+ cells were cultured in 1x Schneider’s *Drosophila* media (GIBCO) supplemented with 10% FBS (GIBCO), penicillin–streptomycin solution (HyClone) in 25-cm^2^ T-flasks (Thermo Fisher Scientific) at 27°C. The cells (∼8 × 10^5^) were seeded in a six-well culture plate and 24 h later were transiently cotransfected with *pUASp-Su(var)2-10-WT::GFP* (Bence et al, 2024), *pUASp-Su(var)2-10-CTD2mut::GFP*, and *pMT-Gal4* using the TransIT-Insect (MIR 6100; Mirus Bio) transfection reagent based on the manufacturer’s recommendations. S2R+ cells were transfected with 2,500 ng of each plasmid. Twenty-four hours after transfection, 0.5 mM of CuSO_4_ was added to the cells to induce Gal4 expression from the metallothionein promoter. 24 h after induction, cells were treated with 100 nM bafilomycin A1 (B1793; Sigma-Aldrich) and incubated for 4 h. Cell pellets were stored at −80°C.

### Immunoprecipitation

Extracts were prepared from 0.5 ml of 3- to 7-d-old whole flies from *da-Gal4>UAS-myr-tdTomato* and *da-Gal4>UAS-mCherry::Atg8a* genotypes. Flies were homogenized in 0.5 ml extraction buffer (50 mM Tris, pH = 7.4, 150 mM NaCl, 2 mM EDTA, 0.5% Nonidet P-40, 5% glycerol, Pierce Protease Inhibitor Tablet [A32963; Thermo Fisher Scientific], and Halt Protease and Phosphatase Inhibitor Cocktail [78442; Thermo Fisher Scientific]) on ice in a Potter homogenizer. After centrifugation (15,000*g*, 15 min, 4°C), the protein concentration in the supernatant was measured with Pierce Bradford Plus reagent (23238; Thermo Fisher Scientific). 5 mg extract was precleared by rotating with 50 μl of Dynabeads M-280 Streptavidin (11206D; Thermo Fisher Scientific) for 1 h at 4°C to absorb bead-binding nonspecific proteins and immunoprecipitated using 50 μl of RFP-Trap Magnetic Agarose (rtma, ChromoTek) for another 1 h at 4°C. Subsequently, beads were washed with extraction buffer three times for 10 min each. Beads were eluted in 20 μl of 2x Laemmli sample buffer without DTT for 5 min at 100°C and diluted and supplemented with DTT. Half of the eluted sample was loaded on gels.

### GST pull-down

GST, GST-Su(var)2-10 WT, and CTD2mut recombinant proteins were produced and purified according to Bence et al (2024). In vitro SUMOylation of Su(var)2-10 was also performed according to Bence et al (2024). HA-Atg8a was synthesized by in vitro transcription/translation from 250 ng PCR product in 25 μl reaction mix (PURExpress In Vitro Protein Synthesis Kit, New England Biolabs). 10 μg GST or GST-tagged proteins were bound to 20 μl Glutathione Sepharose 4B beads (GE Healthcare) and incubated with 5 μl purified HA-Atg8a or 5 μl PURExpress reaction mixture for 2 h at 4°C in binding buffer (50 mM Hepes, pH 7.5, 150 mM NaCl, 2 mM MgCl_2_, 1 mM EGTA, 1 mM DTT, 0.1% Triton X-100, 0.5 mg/ml BSA). After washing three times with the same buffer, the proteins were eluted by incubating the beads with 50 μl 2x SDS–PAGE buffer for 5 min at 95°C.

### Western blotting

S2 cell pellets were lysed on ice for 30 min in three times packed volume RIPA buffer (50 mM Tris, pH = 8, 150 mM NaCl, 1% Nonidet P-40, 0.5% sodium deoxycholate, 0.1% SDS, Pierce Protease Inhibitor Tablet [A32963; Thermo Fisher Scientific], Halt Protease and Phosphatase Inhibitor Cocktail [78442; Thermo Fisher Scientific]) and cleared by centrifugation at 4°C. Protein concentration in the supernatant was measured with Pierce Bradford Plus reagent (23238; Thermo Fisher Scientific). 5 μg total protein was loaded onto 8% polyacrylamide gels. L3-stage homozygous larvae from the *Su(var)2-10*^*MI03442*^/*CyO-GFP* and *w*^*1118*^ genotypes were collected from black agar plates after synchronized egg laying and homogenized in 6 μl Laemmli buffer per μl larva with a pipette tip, then with a motor pestle for 20 s.

For Drpr Western blotting, 100 wings were collected and homogenized in 50 μl RIPA buffer (50 mM Tris, pH = 8, 150 mM NaCl, 5 mM EDTA, 1% Triton X-100, 0.5% sodium deoxycholate, 0.1% SDS, Pierce Protease Inhibitor Tablet (A32963; Thermo Fisher Scientific)) for 2 x 1 min with a motor pestle and cleared by centrifugation at 4°C. For STAT92E::GFP::FLAG detection, four heads per sample were collected in microcentrifuge tubes on dry ice and homogenized in 50–50 μl 1x Laemmli buffer containing 100 mM DTT with a motor pestle for 1 min.

For Drpr Western blotting, for STAT92E::GFP::FLAG detection in a 20 μl sample, 5 μl extract was loaded on 8% polyacrylamide gels. 25 μl GST pull-down eluate was loaded on a 12% polyacrylamide gel. After overnight blotting on Immobilon-FL PVDF Transfer Membrane (Merck), membranes were blocked with Intercept (TBS) Blocking Buffer (LI-COR) for 1 h at RT. The following primary antibodies were used: rabbit anti-GFP (A-11122; Thermo Fisher Scientific), 1:1,000; rabbit anti-mCherry (NBP2-25157; Novus Biologicals), 1:1,000; mouse anti-Su(var)2-10 (Andreev et al, 2022), 1:500; rabbit anti-HA (H6908; Sigma-Aldrich), 1:1,000; mouse anti-α-tubulin (Developmental Studies Hybridoma Bank [DSHB], AA4.3), 1:1,000; mouse anti-Drpr 8A1 (DSHB), 1:100; rabbit anti-glycogen phosphorylase (Tick et al, 1999), 1:2,500; and rabbit anti-β-actin (PA5-85271; Thermo Fisher Scientific), 1:2,500, diluted in Blocking Buffer and incubated for 1 h at RT. The following secondary antibodies were used: anti-mouse IRDye 800CW (926-32210; LI-COR) and anti-rabbit IRDye 680RD (926-68071; LI-COR), 1:15,000, diluted in Blocking Buffer supplemented with 0.02% SDS and 0.2% Tween-20 and incubated for 1 h at RT. Dried blots were imaged in an Odyssey CLx instrument (LI-COR) and band intensities quantified in Image Studio v6.0.

### Image analysis

The signal intensity coming from the fluorescent images was quantified with Fiji (ImageJ 2.9.0, v1.54h). All quantifications were performed on single optical slices. For assessing *TRE-EGFP*, *10xStat92E enhancer-GFP* and *vir-1-GFP* signal intensity levels in wings, we measured a 174 × 54 pixel area in each case. When quantifying the intensity of the vir-1-GFP signal in the brain, intensity data were generated from both optic lobes within a 225 × 37 pixel area. To determine the level of Su(var)2-10 within the nuclei, we randomly selected 10 glial cells surrounding the AL marked by *repo-Gal4 > NLS-GFP* from each brain (except in cases where fewer cells were found). The nuclei were outlined using freehand selection. To measure *drpr* activity in the wing by using *drpr-Gal4 > UAS-TransTimer* flies, we selected an area of 800 × 200 pixel and measured all the nucleus intensities inside the rectangles. The nuclei were outlined by freehand selection. In *dee7-Gal4 > UAS-TransTimer* brains, we used a 1,300 × 1,300 pixel area to count red puncta. In *dee7-Gal4 > UAS-TransTimer* wings, a 600 × 140 pixel-sized rectangular area was selected for counting red puncta. The nucleocytoplasmic ratio of Stat92E::GFP was measured using Fiji. For each brain, four glial nuclei delineated by Repo staining were outlined by the freehand selection tool in the region bordering the antennal lobe to assess the average pixel intensity of the nuclear GFP signal. This value was divided then by the GFP signal average pixel intensity in the surrounding glial cytoplasm close to the nucleus marked by myr::tdTomato, outlined again using the freehand selection tool. All fluorescent signals detected in the area of interest were normalized to the background with identical area sizes. Western blot bands were quantified by Image Studio 5.2 software (LI-COR). Su(var)2-10 signals were normalized to α-tubulin levels.

### Statistical analysis

Experiments were independently repeated at least two times with differing biological samples, with similar results. No data points were excluded. The quantified results from image analysis are represented by truncated violin plots with median and quartiles containing all data points. RNA and protein measurements are presented as the mean and SD. The statistical analyses were performed with GraphPad Prism 8.0.1 and 10. We first checked the normal distribution within each data group by running the Shapiro–Wilk normality test (α = 0.05). When conducting pairwise comparisons on normally distributed data, we performed unpaired, two-tailed *t* test, whereas for non-normally distributed data, the unpaired, two-tailed Mann–Whitney test was applied. For the comparison of multiple datasets of normal distribution, one-way ANOVA with Holm–Šídák’s test for multiple comparisons correction was used as in Fig 6D. No power calculations were performed. For the experiments, we always used a sample size that gave reproducible results, similar to a relevant article (Lu et al, 2017). Western blot and qRT-PCR results were evaluated with n = 3 or 4 biological replicates.

## Supplementary Material

Reviewer comments

## Data Availability

All data needed to evaluate the conclusions in this study are present in the article and its Supplementary Information. Raw image files are deposited on Zenodo: Vincze et al (2025). Source data accompany this article. All materials, *Drosophila* stocks, and related information are available from the corresponding authors upon reasonable request. The research presented here uses the species *D. melanogaster* for which no ethical approval is required in the HUN-REN Biological Research Center, Institute of Genetics, or by Hungarian authorities. Maintenance of transgenic *Drosophila* melanogaster at the Institute is regulated by license No. BGMF/867-9/2022 of the Gene Technology Authority Registry, Ministry of Agriculture of the Hungarian Government.
