# Supplementary material for: Selective autophagy fine-tunes Stat92E activity by degrading Su(var)2-10/PIAS in Drosophila glia
Source: Life Sci Alliance. 2026 Jan 7;9(3):e202503375. doi: 10.26508/lsa.202503375 (PMC12780286; doi:10.26508/lsa.202503375)
Supplement: Supplementary file 2 [file LSA-2025-03375_TableS1.docx]

Supplementary Tables for

**Selective autophagy fine-tunes Stat92E activity by degrading Su(var)2-10/PIAS in *Drosophila* glia**

Virág Vincze^1,2^, Zsombor Esküdt^1,3,4^, Erzsébet Fehér-Juhász^1^, Aishwarya Sanjay Chhatre^1,2^, András Jipa^1^, Anna Rita Galambos^1^, Dalma Feil-Börcsök^1^, Gábor Juhász^1,6^* and Áron Szabó^1^*

Supplementary Table 1. List of studied autophagy genes and their functions

| **autophagy step** | **complex/role** | **gene** | **autophagy specific?** | **other roles** |
| --- | --- | --- | --- | --- |
|  |  |  |  |  |
| initiation | initiation complex | *Atg1* | no | ER-to-Golgi trafficking, alternative autophagy |
|  |  | *Atg13* | no | autophagy-dependent cell death, pathogen control |
|  |  | *Atg101* | yes | not known |
| autophagosome formation | Atg8a ligase complex | *Atg8a* | no | LAP |
|  |  | *Atg16* | no | LAP |
|  |  | *Atg5* | no | LAP |
|  |  |  |  |  |
| cargo recognition and degradation | selective autophagy receptor/autophagic cargo protein | *ref(2)P* | no | TOR kinase, Nrf2, NFkB signaling |

Please see ([Figueras-Novoa et al, 2024](#bibRef06a7b6fd4d16c4765bb9bb5d0750f7ced)), ([Komatsu, 2022](#bibRef0920c3d57016b4d9bace10506532ae54e))  and ([Wang & Kundu, 2017](#bibRef088375f25da3a465b83dd23a5264f7134))  for more information on their canonical versus non-canonical function.

References

Figueras-Novoa C, Timimi L, Marcassa E, Ulferts R, Beale R (2024) Conjugation of ATG8s to single membranes at a glance. *J Cell Sci* 137: jcs261031. doi:10.1242/jcs.261031.

Komatsu M (2022) p62 bodies: Phase separation, NRF2 activation, and selective autophagic degradation. *IUBMB Life* 74: 1200–1208. doi:10.1002/iub.2689.

Wang B, Kundu M (2017) Canonical and noncanonical functions of ULK/Atg1. *Curr Opin Cell Biol* 45: 47–54. doi:10.1016/j.ceb.2017.02.011.
