# Supplementary material for: Selective autophagy fine-tunes Stat92E activity by degrading Su(var)2-10/PIAS in Drosophila glia
Source: Life Sci Alliance. 2026 Jan 7;9(3):e202503375. doi: 10.26508/lsa.202503375 (PMC12780286; doi:10.26508/lsa.202503375)
Supplement: Supplementary file 4 [file LSA-2025-03375_TableS2.docx]

Supplementary Tables for

**Selective autophagy fine-tunes Stat92E activity by degrading Su(var)2-10/PIAS in *Drosophila* glia**

Virág Vincze^1,2^, Zsombor Esküdt^1,3,4^, Erzsébet Fehér-Juhász^1^, Aishwarya Sanjay Chhatre^1,2^, András Jipa^1^, Anna Rita Galambos^1^, Dalma Feil-Börcsök^1^, Gábor Juhász^1,6^* and Áron Szabó^1^*

Supplementary Table 2. Immunity-related gene reporters studied in the wing nerve

| **Reporter with the gene of interest** | **Baseline signal** | | **Signal 3 days after injury** | |
| --- | --- | --- | --- | --- |
|  | **Epithelium** | **Nerve** | **Epithelium** | **Nerve** |
| *AttacinA-GFP* | No | No | Yes | No |
| *Drosocin-GFP* | No | No | No | No |
| *Metchnikowin-GFP* | No | No | Yes | No |
| *Mmp-1-GFP* | No | No | No | No |
| *vir-1-GFP* | Low | Low | Low | Yes |

References

Figueras-Novoa C, Timimi L, Marcassa E, Ulferts R, Beale R (2024) Conjugation of ATG8s to single membranes at a glance. *J Cell Sci* 137: jcs261031. doi:10.1242/jcs.261031.

Komatsu M (2022) p62 bodies: Phase separation, NRF2 activation, and selective autophagic degradation. *IUBMB Life* 74: 1200–1208. doi:10.1002/iub.2689.

Wang B, Kundu M (2017) Canonical and noncanonical functions of ULK/Atg1. *Curr Opin Cell Biol* 45: 47–54. doi:10.1016/j.ceb.2017.02.011.
