# Supplementary material for: Selective autophagy fine-tunes Stat92E activity by degrading Su(var)2-10/PIAS in Drosophila glia
Source: Life Sci Alliance. 2026 Jan 7;9(3):e202503375. doi: 10.26508/lsa.202503375 (PMC12780286; doi:10.26508/lsa.202503375)
Supplement: Supplementary file 10 [file LSA-2025-03375_TableS3.docx]

Supplementary Tables for

**Selective autophagy fine-tunes Stat92E activity by degrading Su(var)2-10/PIAS in *Drosophila* glia**

Virág Vincze^1,2^, Zsombor Esküdt^1,3,4^, Erzsébet Fehér-Juhász^1^, Aishwarya Sanjay Chhatre^1,2^, András Jipa^1^, Anna Rita Galambos^1^, Dalma Feil-Börcsök^1^, Gábor Juhász^1,6^* and Áron Szabó^1^*

Supplementary Table 3. List of primers used in this study

|  |  | forward primer | reverse primer |
| --- | --- | --- | --- |
| ***vir-1* qPCR primers** | *vir-1 AB* | CGACATTGATGAGCCCAAGTCCA | GGGTGCGCTGGTGTGAAGAT |
|  | *vir-1 E* | AGAGGTGCCATCATTTCCACAAC | GGGTGCGCTGGTGTGAAGAT |
|  | *vir-1 ABCGH* | GTACCATCACGCCCTCAGCC | AGACGGCGGAAGAGATCATCG |
|  |  |  |  |
| ***drpr* qPCR primers** | *drpr* | TGTGATCATGGTTACGGAGGAC | CAGCCGGGTGGGCAA |
|  |  |  |  |
| ***RpL32* qPCR primers** | *RpL32* | TGCTAAGCTGTCGCACAAATGGC | CGATCCGTAACCGATGTTGGGC |
|  |  |  |  |
| ***2xmApple-Atg8a* cloning primers** | *Apple1* | gaatacaagaagagaactctgaatagggaattgggaattcATGGTGAGCAAGGGCGAGGA | TTGCTCACCATgcccgagcctcctcctttacccttgtacagctcgtccatgccg |
|  | *Apple2* | tgtacaagggtaaaggaggaggctcgggcATGGTGAGCAAGGGCGAGGA | ATacctcctccgcggccgcctccaccacttcctttgcccttgtacagctcgtccatgccg |
|  | *Atg8a* | ggcaaaggaagtggtggaggcggccgcggaggaggtATGAAGTTCCAATACAAGGAGGAG | ttccttcacaaagatcctctagaggtaccctcgagttaGCCGTAAACATTCTCATCGGAG |

References

Figueras-Novoa C, Timimi L, Marcassa E, Ulferts R, Beale R (2024) Conjugation of ATG8s to single membranes at a glance. *J Cell Sci* 137: jcs261031. doi:10.1242/jcs.261031.

Komatsu M (2022) p62 bodies: Phase separation, NRF2 activation, and selective autophagic degradation. *IUBMB Life* 74: 1200–1208. doi:10.1002/iub.2689.

Wang B, Kundu M (2017) Canonical and noncanonical functions of ULK/Atg1. *Curr Opin Cell Biol* 45: 47–54. doi:10.1016/j.ceb.2017.02.011.
