# Supplementary material for: Selective autophagy fine-tunes Stat92E activity by degrading Su(var)2-10/PIAS in Drosophila glia
Source: Life Sci Alliance. 2026 Jan 7;9(3):e202503375. doi: 10.26508/lsa.202503375 (PMC12780286; doi:10.26508/lsa.202503375)
Supplement: Reviewer comments [file LSA-2025-03375_review_history.pdf]

Selective autophagy fine-tunes Stat92E activity by degrading Su(var)2-10/PIAS in Drosophila glia

Virág Vincze, Zsombor Esküdt, Erzsébet Fehér-Juhász, Aishwarya Chhatre, András Jipa, Anna Galambos, Dalma Feil-Börcsök, Melinda Bence, Gabor Juhasz, and Áron Szabó
DOI: https://doi.org/10.26508/lsa.202503375

Corresponding author(s): Áron Szabó, HUN-REN Szegedi Biológiai Kutatóközpont

Review Timeline:

Submission Date:	2025-04-30
Editorial Decision:	2025-05-09
Revision Received:	2025-10-16
Editorial Decision:	2025-11-20
Revision Received:	2025-11-29
Accepted:	2025-12-03

Scientific Editor: Tim Fessenden

Transaction Report:

(Note: With the exception of the correction of typographical or spelling errors that could be a source of ambiguity, letters and reports are not edited. The original formatting of letters and referee reports may not be reflected in this compilation.)

Please note that the manuscript was reviewed at *Review Commons* and these reports were taken into account in the decision-making process at *Life Science Alliance.*

Reviews

Review #1

1. Evidence, reproducibility and clarity:

Summary:

Regulation of immune pathway responses in glia is critical after nervous system injuries.
The authors use the nerve fibers in the Drosophila wing as a model system to further analyze molecular mechanisms of glial responses with a focus on the regulation of the STAT92E protein, the single STAT family protein in Drosophila, which in glial injury responses has previously been shown to be independent of the canonical Domeless receptor / JAK kinase pathway.

Here, the authors show convincingly that STAT92E activation depends on the selective autophagic degradation of the SUMOligase Su(var)2-10/PIAS in the absence of elevated bulk autophagy. IF and IP experiments indicated that direct or indirect interactions with Atg8 may drive this selective autophagy of Su(var)2-10/PIAS and that its SUMOylation activity appears to promote its degradation.

Further observations show that STAT92E in this context does not result in elevated expression of the glial phagocytic Draper receptor and instead yields elevated vir-1 expression with unknown consequences for neuronal health.

All key conclusions in the paper are well supported by experimental evidence and careful quantification.

Major comments:

Figure 6E seems to indicate that a subset of Su(var)2-10/PIAS isoforms may bind to ATG8 (directly or indirectly).
This leads to the straightforward prediction that this subset should be differentially affected by the selective autophagy at the center of the manuscript. That could be tested to strengthen that point.

Minor comments:

- in Fig S1B,C the colocalization between GFP reporters for STAT92E and AP-1 activity and glia marker does not seem convincing, indicating other cell types may be expressing them as well.
- p.7 Instead of "Su(var)2-10 is mainly nuclear due to its transcriptional repressor and chromatin organizer functions" It may be better to say" .. .consistent with its transcriptional repressor and chromatin organizer functions"
- It is not clear whether the differences in Su(var)2-10/PIAS accumulation between Atg16 and Atg101 RNAi indicate functional differences of blocking autophagy at different stages or simply differences in RNAi efficiency (Atg16) versus the Atg101 mutant.

2. Significance:

The manuscript convincingly shows that autophagic degradation is an important component of the regulation of STAT92E, an important transcriptions factor for glial responses to nerve injuries. That is a novel observation that will be of interest to experts in the field of autophagy and its roles in brain homeostasis.

In addition. some other interesting initial observations are reported, but without much follow up that could have significantly strengthened the paper:

- STAT92E-dependent glial upregulation of vir-1, but not Draper, is shown, but consequences for glial functions in nerve injury are not tested.
- experiments indicate a role for Su(var)2-10/PIAS SUMOylation activity in tis autophagic degradation, but it is not clear whether the critical substrata Su(var)2-10/PIAS itself or another protein.
- binding of Su(var)2-10/PIAS to ATG8 is indicated, but no in vitro experiment performed to test whether this is direct and perhaps SUMOylation dependent.

Review #2

1. Evidence, reproducibility and clarity:

Summary:

The manuscript by Vincze et al. explores the regulatory mechanisms of Stat92E in glial reactivity following axonal injury. Utilizing a wing injury model in Drosophila, the study demonstrates the role of autophagy in regulating Stat92E expression in glia during injury. Through genetic and biochemical assays, the authors reveal that autophagy facilitates the degradation of Su(var)2-10, a negative regulator of Stat92E, thereby enabling the activation of this pathway. Overall, this study highlights a crucial role for autophagy in glial immunity during axonal injury.

Major comments:

- Are the claims and the conclusions supported by the data or do they require additional experiments or analyses to support them?

The working hypothesis is that upon injury, Su(var)2-10 is degraded by autophagy and, as a consequence, Stat92E induces vir-1 expression.
Could the authors clarify why do Stat92E levels increase upon injury? Does Stat92E stability increase upon ATG mediated Su(var)2-10 degradation? Or does it expression/nuclear translocation change?
Also, since Su(var) levels increase upon ATG RNAi, independently of injury, do ATG levels increase upon injury? It does not seem to be the case from Fig 6D, but then, if the ATG levels do not increase, how to explain the injury mediated effects of Su(var)2-10?
Su(var)2-10 levels in control and injured wings are different between ATG18RNAi and ATG101 mutant (Fig 5). Could the authors explain the rational for using two ATG mutants? and the meaning of this difference? Also, why comparing data using the RNAi approach and a mutation?
Fig 6 What is the relevance of the Atg8, Sumo and Su(var)2-10 colocalization at puncta, since there is a lot of colocalization outside the puncta and also lots of Su(var)2-10 or Atg8 labeling that does not colocalize?
The statement made in the first sentence of the discussion is very strong: 'we have uncovered an activation mechanism for Stat92E', without sufficient supporting evidence.
- Please request additional experiments only if they are essential for the conclusions. Alternatively, ask the authors to qualify their claims as preliminary or speculative, or to remove them altogether.

Could the authors validate (some) expression data by in situ hybridization experiments?
Could the authors validate the RNAi lines molecularly (or refer to published data on these lines?
- If you have constructive further reaching suggestions that could significantly improve the study but would open new lines of investigations, please label them as "OPTIONAL".
- Are the suggested experiments realistic in terms of time and resources? It would help if you could add an estimated time investment for substantial experiments.

Clarifying the role of Su(var)2-10 on Stat92E would benefit to the presented work. Does Atg8-Su(var)2-10 binding affect Stat92E accumulation, expression, translocation to the nucleus?
Some of these experiments could be obtained in S2 cell transfection assays, if too complex in vivo.
Also, what happens to the axons in the mutant conditions described in the manuscript? This would higher the impact of the work, but would require in vivo work with fly stocks containing several transgenes.
- Are the data and the methods presented in such a way that they can be reproduced?

It has been published that Draper is involved in the response to injury in the adult wing nerve. See for example Neukomm et al (2014). The authors should discuss how this fits with their hypothesis and data. In this respect, Fig S4B, which should support the hypothesis, should be improved. It is rather hard to interpret it.
- Are the experiments adequately replicated and statistical analysis adequate?

Yes

Minor comments:

- Specific experimental issues that are easily addressable.

Rubicon is also a negative regulator of autophagy (doi:10.1038/s41598-023-44203-6). in (Fig2 B, D) we have a higher GFP intensity in both uninjured and injured, and the difference between Injured/uninjured is less significant compared to control. It is possible that Rubicon KD causes more autophagy leading to a higher activation of Stat92E even in control. I wouldn't take the results as a proof of canonical autophagy implication and not LC3-associated phagocytosis
The rationale for using both repoGal4 and repoGS is unclear. If, as mentioned, the goal is to avoid developmental defects, repoGS should be consistently used. Especially I don't understand how both were utilized to knock down the same genes, such as Atg16.
In the third paragraph of the introduction, I am confused whether Stat92E regulates drpr of the reverse?
- Are prior studies referenced appropriately?

Published work should be acknowledged properly.
I cannot find the evidence for vir-1 being expressed in glia and target of Gcm in the refences that have been cited.

The presence of a Stat92E binding site on the vir-1 promoter has already bene described in the paper from Imler and collaborators, Nature immunology 2005. Actually, if this site is present in their transgenic line, it would help the authors strengthen the argument that Stat92E has a direct role on vir1 (for which they make a very strong statement in the discussion, with no direct evidence).
- Are the text and figures clear and accurate?
- Do you have suggestions that would help the authors improve the presentation of their data and conclusions?

In the Fig S2D, I do not see a lot of GFP+ (Glia) cells. I see more Atg8a in injured 3 dpi regardless of colocalization with glia.
The quantification of the signals is made in a specific region of the wing, I guess throughout the nerve thickness. This could be represented more carefully in a schematic and It would also help defining colocalization in the first figure, by using a transverse section.
A number of ATG genes are considered in the manuscript, but the rational for using them is not always clear. Showing a schematic would help clarify this. Fig 7 is not cited and its legend is very short.

Clarify the color coding in Fig S1E

What is the tandem tagged autophagic fly reporter in fig S2D?

Add a schematic on the vir-1 isoforms.

Fig S6B and Fig 5 relate on the levels of Su(var)2-10 upon Atg16 RNAi, but the scale is not the same, why?

2. Significance:

- *General assessment:* provide a summary of the strengths and limitations of the study. What are the strongest and most important aspects? What aspects of the study should be improved or could be developed?
The manuscript by Vincze et al. investigates the regulatory mechanisms of Stat92E in glial reactivity following axonal injury. This research addresses a significant topic relevant to neuroinflammatory conditions in humans, such as neurodegenerative diseases. Utilizing a wing injury model in Drosophila, the study identifies a novel upstream regulatory mechanism of Stat92E. Specifically, after axonal injury, autophagy facilitates the degradation of Su(var)2-10, a negative regulator of Stat92E in glia. This process enables a non-canonical activation of the JAK-STAT pathway, leading to the induction of downstream target genes, such as Vir-1, highlighted in this study. Altogether, the manuscript advances our understanding of the glial response to neuronal damage, building on previous work by this group and others. Notably, it highlights progress in the role of both autophagy machinery and JAK-STAT pathway in this context.
- *Limitations and possible improvements:*
A more mechanistic analysis will higher the impact of the findings.
Clarifying the role of Su(var)2-10 on STAT92E would benefit to the presented work. Does Atg8-Su(var)2-10 binding affect STAT92E accumulation, expression, translocation to the nucleus?
Also, what happens to the axons in the mutant conditions described in the manuscript?
- *Advance:* compare the study to the closest related results in the literature or highlight results reported for the first time to your knowledge; does the study extend the knowledge in the field and in which way? Describe the nature of the advance and the resulting insights (for example: conceptual, technical, clinical, mechanistic, functional,...).
The work provides a conceptual advance in the field by assessing the role of ATG genes and a novel pathway linked

to STAT and vir-1.
- *Audience:* describe the type of audience ("specialized", "broad", "basic research", "translational/clinical", etc...) that will be interested or influenced by this research; how will this research be used by others; will it be of interest beyond the specific field?
A broad audience working on neurodegeneration will be interested in the described work.
- Please define your field of expertise with a few keywords to help the authors contextualize your point of view. Indicate if there are any parts of the paper that you do not have sufficient expertise to evaluate.
Neural development and the transcriptional mechanisms involved to the process.

Review #3

1. Evidence, reproducibility and clarity:

In this study the authors explore a potential role for STAT92E and Su(var)2-10 in glial responses to injury in the adult Drosophila wing. The major claims are that canonical autophagy and not LAP sustains STAT92E signaling after in jury. The negative regulator STAT92E is removed by selective autophagy, but this is not ref(2)p/p62 (perhaps). Glial draper expression is not upregulated and Draper accumulation is not apparently a prerequisite for efficient debris clearance in the wing. Su(var)2-10 is an autophagic cargo, mediator of STAT92E-dependennt transcription; and interacts with Atg8a, perhaps sumoylating targets. In general, the model is reasonable, but the data do not support the conclusions, and the quality of the data needs improvement before firm conclusions can be reached. Concerns include:

1. The claim that the negative regulator of Stat92E signaling is removed by selective autophagy, involving selective autophagy receptors different from/in addition to Ref(2)P/p62 is not convincingly shown. This claim probably needs to be softened.
2. The reporter that was used (10xSTAT92E-eGFP) is not a dynamic reporter of STAT92E activity. It accumulates in glia and is highly stable. The appropriate reporter to look at dynamic changes would be 10XSTAT92E-dGFP, which has a degradable (unstable) GFP that is required to see dynamic changes even in the CNS. All of the claims about STAT92E regulation use this reporter, so they are questionable.
3. The claim that glial drpr is not upregulated by wing injury and drpr accumulation is not apparently a prerequisite for efficient debris processing within the wing is weak. First, they did not stain for Draper using antibodies, rather they used expression constructs. Dee7 is a promoter that was found to be injury activated in the CNS (were they able to replicate that result? I did not receive the supplemental data), but it might not be the crucial regulator in the periphery. The MIMIC line that was converted is better, but might not represent the full spectrum of regulatory events at the draper locus. Finally, they never actually test for endogenous RNA changes, or use the antibody on westerns. Their lack of evidence is not as compelling as it could be.
4. The authors claim autophagy contributes to glial reactive states in part by acting on JAK-STAT pathway via regulation of Stat92E. They did not investigate other potential STAT92E targets. Does Atg16 knockdown alter STAT92E expression? Apparently Vir1 is still upregulated in the absence of Atg16 following injury, but they don't show STAT92E changes.
5. The authors claim Su(var)2-10 is an autophagic cargo. They should better characterize Su(var)2-10 degradation and its regulation, and image quality needs to be improved (better images, merged examples, and clearer indication of what they are highlighting. There are many arrows in figures that I don't know what they are pointing to. Much of the labeling in Fig 1 (and others) looks like axons. Could TRE-GFP be turned on in neurons? How did they discriminate?
6. The authors claim interaction of Su(var)2-10 with Atg8a in the nucleus and cytoplasm can trigger autophagic breakdown, involving Su(var)2-10 SUMOylation. The paper would benefit from showing direct SUMOylation of Su(var)2-10 after injury. Is there any way to examine this in vivo?
The authors state in discussion "we find that draper is highly expressed in wing nerve glia already in uninjured conditions and it is not further induced by wing transection - indicating high phagocytic capacity in wing glia ... axon debris clearance takes substantially longer in the wing nerve than in antennal lobe glomeruli, thus draper levels may not readily predict actual phagocytic activity in glia". However, they never actually assess this in their experiments. All the conclusions about Draper are made from promoter fusions of integrated reporters, which are imperfect. This conclusion cannot be made.
Both STAT92E and Jun are activated by a stress response. Could this be a stress response to disrupting autophagy that is somehow enhance by injury?

Minor:

I don't think that "glially" is a word.

2. Significance:

Based on the quality of the data, it is hard to consider this manuscript having made a major step forward. A significant amount of work needs to be done to firm up the conclusions. In its present form, the major contributions are the identification vir-1 as upregualted (maybe) and a potential role for autophagy.

May 9, 2025

Re: Life Science Alliance manuscript #LSA-2025-03375-T

Dr. Áron Szabó
HUN-REN Biological Research Center
Institute of Genetics
Temesvári krt 62
Szeged 6726
Hungary

Dear Dr. Szabó,

Thank you for transferring your manuscript entitled "Selective autophagy sets Stat92E activity by degrading Su(var)2-10 during glial injury signalling" to Life Science Alliance. We invite you to submit a revised manuscript addressing the Reviewer comments as stipulated in your revision plan from Reviewer Commons.

To upload the revised version of your manuscript, please log in to your account: https://lsa.msubmit.net/cgi-bin/main.plex

You will be guided to complete the submission of your revised manuscript and to fill in all necessary information. Please get in touch in case you do not know or remember your login name.

While you are revising your manuscript, please also attend to the below editorial points to help expedite the publication of your manuscript. Please direct any editorial questions to the journal office.

The typical timeframe for revisions is three months. Please note that papers are generally considered through only one revision cycle, so strong support from the referees on the revised version is needed for acceptance.

When submitting the revision, please include a letter addressing the reviewers' comments point by point.

Thank you for this interesting contribution to Life Science Alliance. We are looking forward to receiving your revised manuscript.

Sincerely,

Tim Fessenden
Scientific Editor
Life Science Alliance

A. THESE ITEMS ARE REQUIRED FOR REVISIONS

-- A letter addressing the reviewers' comments point by point.

-- An editable version of the final text (.DOC or .DOCX) is needed for copyediting (no PDFs).

-- High-resolution figure, supplementary figure and video files uploaded as individual files: See our detailed guidelines for preparing your production-ready images, https://www.life-science-alliance.org/authors

-- Summary blurb (enter in submission system): A short text summarizing in a single sentence the study (max. 200 characters including spaces). This text is used in conjunction with the titles of papers, hence should be informative and complementary to the title and running title. It should describe the context and significance of the findings for a general readership; it should be written in the present tense and refer to the work in the third person. Author names should not be mentioned.

-- By submitting a revision, you attest that you are aware of our payment policies found here: https://www.life-science-alliance.org/copyright-license-fee

B. MANUSCRIPT ORGANIZATION AND FORMATTING:

Full guidelines are available on our Instructions for Authors page, https://www.life-science-alliance.org/authors

We encourage our authors to provide original source data, particularly uncropped/-processed electrophoretic blots and spreadsheets for the main figures of the manuscript. If you would like to add source data, we would welcome one PDF/Excel-file per figure for this information. These files will be linked as supplementary "Source Data" files.

IMPORTANT: It is Life Science Alliance policy that if requested, original data images must be made available. Failure to provide original images upon request will result in unavoidable delays in publication. Please ensure that you have access to all original microscopy and blot data images before submitting your revision.

Manuscript number: LSA-2025-03375-TR

Corresponding author(s): Áron, Szabó; Gábor, Juhász

- We thank the reviewers for their useful suggestions regarding how to improve our manuscript.

- Reviewer 3 declared that s/he did not find and evaluate the provided Supplementary Materials. As a result, many of her/his criticisms seem invalid because the requested data, validations etc. were already there in the Supplementary Figures and Tables.

- To avoid confusion, we renamed the transgene that is commonly used as a readout for STAT-activated transcription from *10xStat92E-GFP* to *10xStat92E enhancer-GFP* (please see comments by Reviewer 2 that show how easily one can think that Stat92E protein levels go up because of the misleading name of this transgene).

- One co-author, Martin Csordós was among the authors by mistake. Although first considered, his contribution was not included in either the original or the current manuscript version, so we removed his name from the revised version with his permission.

- We prefer to use colour coding in our responses to Reviewer comments rather than splitting the responses to queries in separate sections, because many of our answers contain both experimental answers to reviewer questions and explanations why we think that no additional analyses are necessary. Data already provided in the original submission but perhaps overlooked by Reviewers has white background in our responses.

Description of executed revisions

Description of analyses that authors preferred not to carry out

Reviewer comments and point-by-point response of the authors

Reviewer 1

Major comments:

R1/1. "Figure 6E seems to indicate that a subset of Su(var)2-10/PIAS isoforms may bind to ATG8 (directly or indirectly).
This leads to the straightforward prediction that this subset should be differentially affected by the selective autophagy at the center of the manuscript. That could be tested to strengthen that point. "

Response:

The Atg8a-binding subset of Su(var)2-10/PIAS isoforms could indeed be differentially affected by selective autophagy. To test this, we have analyzed Su(var)2-10 isoform abundance in vivo on western blots of Atg8a$^{K48A/Y49A}$ (Atg8a^{LDS}) mutant whole fly extracts with an anti- Su(var)2-10 antibody. Whole fly extracts were used for immunoprecipitation in Fig. 6E (now Fig. 7E) as well.

We did not find a statistically significant increase in the abundance of different Su(var)2-10 isoforms although a clear trend towards higher levels in the case of bands"a" „b" and „c" was observed (please see Fig. 1 for Reviewers below). The cell type specificity of Su(var)2-10 isoform degradation by autophagy may explain this result, because we do see glial upregulation of Su(var)2-10 by brain immunostaining (Fig. 5.). In other words, Su(var)2-10 interaction with Atg8a may occur only in a subset of tissues such as glia and was still detected by immunoprecipitation. Su(var)2-10 from other tissues in the body may mask the altered Su(var)2-10 abundance in the Atg8a^{LDS} mutant on western blot of whole-body lysates.

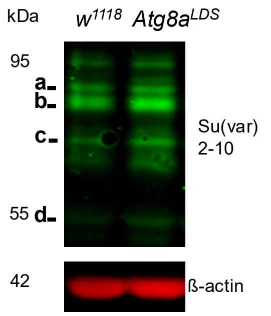

Figure 1 for Reviewers. *Su(var)2-10 immunoblot of whole fly extracts identifies three major bands (a, b and c) in control (w^{1118}) and Atg8a^{LDS} animals. β-actin immunoblot is shown for loading control.*

Minor comments:

R1/2. " in Fig S1B,C the colocalization between GFP reporters for STAT92E and AP-1 activity and glia marker does not seem convincing, indicating other cell types may be expressing them as well."

Response:

The overlap between glia labelling and STAT92E and AP-1 transcriptional readout reporter expression is indeed not complete. First of all, epithelial cells in the wing display both STAT92E and AP-1 activity, even in uninjured conditions when glial expression of these reporters is not yet observed. We previously indicated transcriptional reporter activity outside of the wing nerve with arrowheads, but now the epithelium is labeled with text and the regions containing wing nerve glia are outlined in all relevant figures.

The fiber-like reporter expression after injury in the wing nerve could correspond to either glia or axons[1–3]. Glia in the wing nerve have a filament-like appearance resembling axons in confocal images - even glial nuclei are flat/elongated[1]. Importantly, STAT92E enhancer-driven GFP also labels the nucleus in expressing cells, as opposed to glially driven mtdTomato that is membrane-tethered (and thus excluded from the nucleus: please see Fig. S2A,B). We have added new images from injured conditions and corresponding transverse confocal sections of these genotypes to Fig. S2A,B (previously S1B,C) to better illustrate the presence of reporter activity in glia. Of note, TRE-GFP and 10x Stat92E enhancer-GFP are not expressed in neurons because the cell bodies and nuclei of wing vein neurons are not GFP-positive, see Fig. 2C, Figs. S1, S4 in Neukomm et al.[1] and new panels in Fig. S2C,D in this manuscript. We also aimed to explain this better now in the revised manuscript (please see the legend of Fig. S2).

R1/3. "p.7 Instead of "Su(var)2-10 is mainly nuclear due to its transcriptional repressor and chromatin organizer functions" It may be better to say" .. .consistent with its transcriptional repressor and chromatin organizer functions""

Response:

We have modified the manuscript accordingly.

R1/4. "It is not clear whether the differences in Su(var)2-10/PIAS accumulation between Atg16 and Atg101 RNAi indicate functional differences of blocking autophagy at different stages or simply differences in RNAi efficiency (Atg16) versus the Atg101 mutant."

Response:

We have added glia-specific Atg1 (the catalytic subunit of the autophagy initiation complex that also includes Atg101) knockdown experiments that show the same lack of Su(var)2-10 accumulation in uninjured conditions as seen in the Atg101 null mutant (please see Fig. S7C-E). Please note that Atg16-Atg5-Atg12 dependent conjugation of LC3/Atg8a is involved in various vesicle trafficking pathways in addition to autophagy[4–6], alterations of which may perturb baseline Su(var)2-10 levels in uninjured animals.

Significance:

R1/5. "STAT92E-dependent glial upregulation of vir-1, but not Draper, is shown, but consequences for glial functions in nerve injury are not tested."

Response:

We decided to test whether vir-1 or Stat92E are required for two injury-related glial functions: antimicrobial activity and glial motility towards damaged tissue.

Certain antimicrobial peptides (AMPs) such as Attacin C are known to be regulated by both the Stat and NF-κB pathways[7], and AMPs can generally be upregulated in response to brain injury[8,9]. This could mediate pathogen clearance after defense lines such as the epithelium and blood-brain barrier are compromised. We used a Mtk-GFP reporter bearing two consensus STAT binding sites as an AMP readout. After antennal ablation, we did not observe a difference in Mtk-GFP levels among control, vir-1 or Stat92E-deficient and proficient conditions.

We also assessed the recruitment of glial processes into the antennal lobe after olfactory nerve injury in animals with glia-specific Stat92E or vir-1 deficiency. Glial invasion is an adaptive response to axon injury and a first step towards debris clearance[10]. We did not find a glial membrane recruitment defect after antennal ablation upon either Stat92E or vir-1 knockdown. Hence, the role of vir-1 in glial injury responses still remains to be elucidated.

R1/6. "experiments indicate a role for Su(var)2-10/PIAS SUMOylation activity in tis autophagic degradation, but it is not clear whether the critical substrata Su(var)2-10/PIAS itself or another protein."

"binding of Su(var)2-10/PIAS to ATG8 is indicated, but no in vitro experiment performed to test whether this is direct and perhaps SUMOylation dependent."

Response:

We aimed to answer this question in the original manuscript by using a point mutant form of *Su(var)2-10 referred to as CTD2, which is unable to properly autoSUMOylate itself*[12]*, see Fig. 7C,D. CTD2 mutant Su(var)2-10 levels increased in S2 cells transfected with the mutant construct relative to the wild-type, and lysosome inhibition affected the wild-type protein level but not the mutant variant. Importantly, wild-type Su(var)2-10 is present in CTD2 mutant Su(var)2-10-transfected cells, which can still SUMOylate other Su(var)2-10 targets. These thus point to the intrinsic SUMOylation defect of the CTD2 mutant that results in its impaired degradation. It is firmly established that increased Su(var)2-10/PIAS levels repress STAT92E activity*[13]*, mammalian example: Liu et al., 1998*[14]*, indicating that Su(var)2-10 is the critical substrate for autophagy during STAT92E derepression.*

We have further addressed this point and investigated if Su(var)2-10 directly binds to Atg8a by in vitro SUMOylation of GST-Su(var)2-10 and subsequent GST pulldown assay with HA-Atg8a. In vitro SUMOylation reaction with purified GST-Su(var)2-10 and negative controls have been described by our collaborators[12]*. We incubated the resulting SUMOylated GST-Su(var)2-10 and non-SUMOylated counterparts with in vitro transcribed /translated HA-Atg8a, and interactions were tested by GST pulldown and anti-HA western blotting. We could not detect interaction between either SUMOylated or unmodified GST-Su(var)2-10 and HA-Atg8a (please see Fig. 2 for Reviewers below). Therefore we presume that there is no direct interaction between Su(var)2-10 and Atg8a.*

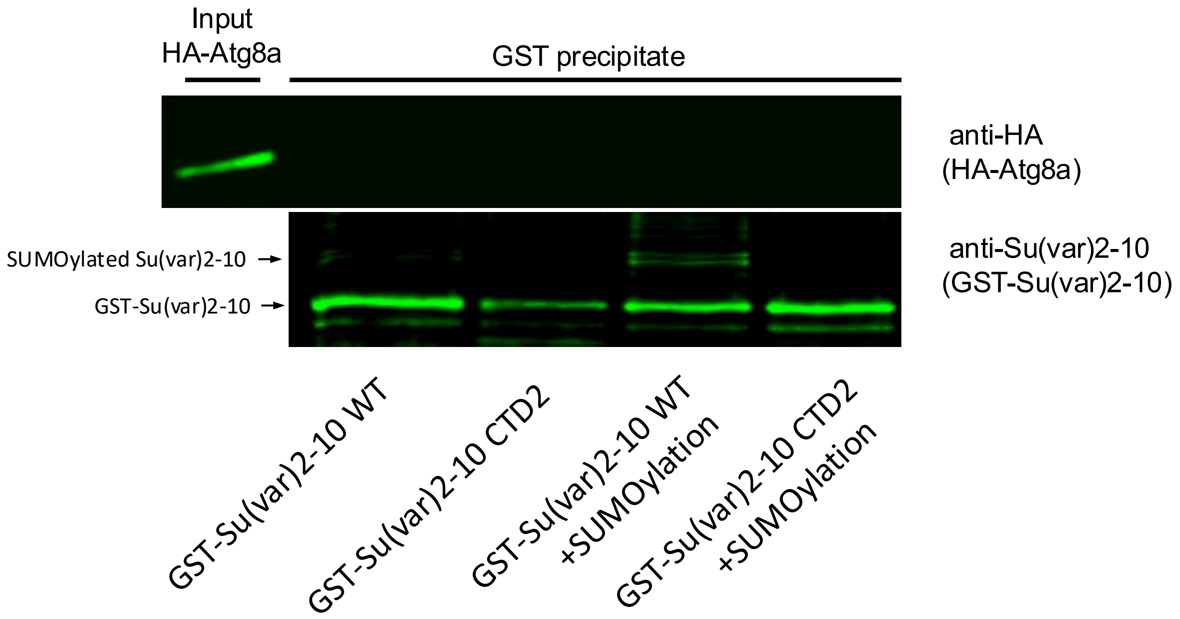

Figure 2 for Reviewers. *Purified GST-Su(var)2-10 was in vitro SUMOylated. Unmodified and SUMOylated wild-type and SUMOylation defective CTD2 variant Su(var)2-10 were incubated with in vitro transcribed /translated HA-Atg8a and interactions were tested by GST pulldown and anti-HA western blotting.*

Reviewer 2

Major comments:

R2/1. *"*The working hypothesis is that upon injury, Su(var)2-10 is degraded by autophagy and, as a consequence, Stat92E induces vir-1 expression.

Could the authors clarify why do Stat92E levels increase upon injury? Does Stat92E stability increase upon ATG mediated Su(var)2-10 degradation? Or does it expression/nuclear translocation change?"

Response:

We did not state in the original manuscript that Stat92E levels increase upon injury - we only used the 10xStat92E enhancer-GFP reporter (we have renamed it as such in our revised manuscript to avoid confusion) that is commonly referred to as 10xStat92E-GFP in the literature[15]*, as a readout for Stat92E-dependent transcription.*

To address the above questions, we have now used a transgenic, endogenous promoter-driven STAT92E::GFP::FLAG protein-protein fusion (https://flybase.org/reports/FBti0147707.htm) to test if STAT92E stability/expression or translocation is altered during injury or upon disruption of selective autophagy. As the $Atg8a^{LDS}$ mutation specifically impairs selective autophagy, we used this mutant and wild-type controls to assess STAT92E::GFP::FLAG abundance on western blots. STAT92E abundance did not change in $Atg8a^{LDS}$ head lysates (Fig. S8A).

To assess STAT92E::GFP::FLAG nuclear translocation as well as stability/expression in tissues, we used $Atg8a^{LDS}$ to perturb STAT92E -dependent transactivation. We visualized glial cell membranes around the antennal lobe of dissected brains after antennal ablation[10] *by membrane-tethered tdTomato, glial nuclei by DAPI/anti-Repo, and STAT92E with the STAT92E::GFP::FLAG fusion transgene. We indeed found an increase in glial STAT92E abundance after injury which was not significant in $Atg8a^{LDS}$ brains (Fig. 6 A,B, S8B). Also, the STAT92E::GFP::FLAG nuclear-cytoplasmic ratio increased in glia in injured brains, and this effect disappeared in $Atg8a^{LDS}$ flies (Fig.6 A,C, S8C,D). This indicates defective nuclear import of STAT92E when selective autophagy is impaired.*

R2/2. *"*Also, since Su(var) levels increase upon ATG RNAi, independently of injury, do ATG levels increase upon injury? It does not seem to be the case from Fig 6D, but then, if the ATG levels do not increase, how to explain the injury mediated effects of Su(var)2-10? "

Response:

We have not seen an effect of injury on the rate of autophagic degradation (flux) using the common flux reporter GFP-mCherry -Atg8a in glia after injury (shown in Fig. S2D, now S3E – not 6D). Also, levels of the typical autophagic cargo p62/Ref(2)P and core autophagy proteins such as Atg12, Atg5, Atg16 do not change after nervous system injury[18] *suggesting that there is no change in general autophagic turnover.*

Similar to the ubiquitin-proteasome system, labelling of the cargo/substrate for degradation is a regulated process in selective autophagy. Dynamic ubiquitylation of a cargo often promotes its autophagic degradation[19]*. We hypothesize that SUMO may fulfil a similar role in labelling cargo for elimination and this may be promoted by injury in the case of Su(var)2-10, which warrants future studies.*

R2/3. "Su(var)2-10 levels in control and injured wings are different between ATG18RNAi and ATG101 mutant (Fig 5). Could the authors explain the rational for using two ATG mutants? and the meaning of this difference? Also, why comparing data using the RNAi approach and a mutation?"

Response:

This issue was also raised in R1/4 and we refer the Reviewer to that section for our new Atg1 knockdown data and explanations.

There is a consensus in the autophagy community that mutants for multiple Atg genes should always be used to ensure that it is indeed canonical autophagy that is affected (because Atg proteins can have non-autophagic roles, as is the case for Atg16 in regulation of phagosome maturation - LAP).

R2/4. "Fig 6 What is the relevance of the Atg8, Sumo and Su(var)2-10 colocalization at puncta, since there is a lot of colocalization outside the puncta and also lots of Su(var)2-10 or Atg8 labeling that does not colocalize? "

Response:

Su(var)2-10 orthologs PIAS1-4 localize to the nuclear matrix and certain foci in the chromatin and may play roles in heterochromatin formation, DNA repair, and repression of transposable elements in addition to transcriptional repression[20–22]*. We show colocalization of Atg8a with similar Su(var)2-10 and SUMO double positive structures in foci, which may relate to the accumulation of SUMO-modified proteins in phase-separated foci also referred to as SUMO glue*[23] *in response to PIAS activity.*

We do not expect a full overlap between Su(var)2-10 and Atg8a labeling for a number of reasons. First, Su(var)2-10 has many different roles that may not be regulated by autophagy. Second, Atg8a+ autophagosomes in the cytoplasm deliver not only indidivual proteins such as Su(var)2-10 for degradation but also many other cellular components. Third, nuclear Atg8a is implicated in the removal of the Sequoia transcriptional repressor from autophagy genes that is unlikely to involve Su(var)2-10[24]*. Now we include these points in the Discussion section.*

R2/5. "The statement made in the first sentence of the discussion is very strong: 'we have uncovered an activation mechanism for Stat92E', without sufficient supporting evidence."

Response:

We have rephrased this section as follows:
„Here we have uncovered that downregulation of a direct repressor of the Stat92E transcription factor, and thus Stat92E activation and nuclear translocation, are mediated by autophagy."

R2/6. "Could the authors validate (some) expression data by in situ hybridization experiments?"

Response:

Unfortunately, in situ hybridization is not feasible in this organ as probes do not penetrate the thick chitin-based cuticule and wax cover of the wing (and the same is true for wing immunostaining). To corroborate reporter-based data, we do provide independent evidence for vir-1 upregulation in the wing after injury via quantitative PCR (qPCR) in Fig. S6C. Similarly, we also analyzed drpr in qPCR using wing material after injury at the same time points. We did not find significant alterations in drpr levels after wing injury (Fig. S5D).

R2/7. "Could the authors validate the RNAi lines molecularly (or refer to published data on these lines?"

Response:

Almost all RNAi lines have already been validated by qPCR, western blot, or immunostaining in Szabo et al., 2023[18] *and other publications*[25–27]*. The only exception is Su(var)2-10*^{JF03384} *and we show that its phenotype is indistinguishable from the validated Su(var)2-10*^{HMS00750} *RNAi line (which causes 95% transcript reduction): it also strongly derepresses STAT activity. These reagents have also*

been widely used in the community (e.g. https://flybase.org/reports/FBal0242556.htm, https://flybase.org/reports/FBal0233496.htm).

R2/8. „Clarifying the role of Su(var)2-10 on Stat92E would benefit to the presented work. Does Atg8-Su(var)2-10 binding affect Stat92E accumulation, expression, translocation to the nucleus? Some of these experiments could be obtained in S2 cell transfection assays, if too complex in vivo."

Response:

As explained in R2/1 (please see earlier), we have used an endogenous promoter-driven STAT92E::GFP::FLAG protein-protein fusion transgene to test if STAT92E stability/expression or translocation is altered upon disruption of selective autophagy (in Atg8a^{LDS} mutant flies).

R2/9. „Also, what happens to the axons in the mutant conditions described in the manuscript? This would higher the impact of the work, but would require in vivo work with fly stocks containing several transgenes."

Response:

We have already published in our previous paper: Szabo et al., 2023[18] that the mutants used in the current study display normal axon morphology. There are only two mutants that we did not test in that paper: Atg8a^{LDS} and our new Atg8a^{null}. We thus examined these remaining two during the revision. Atg8a^{null} in the background of OK371-Gal4, UAS-mCD8::GFP developed deformed wings that could not be analysed, but we already published in the above paper that axons appear normal in Atg8a$^{\Delta4}$, a widely used Atg8a severe hypomorphic mutant allele. Axons in the Atg8a^{LDS} background appeared normal (Fig. S3B).

R2/10. „It has been published that Draper is involved in the response to injury in the adult wing nerve. See for example Neukomm et al (2014). The authors should discuss how this fits with their hypothesis and data. In this respect, Fig S4B, which should support the hypothesis, should be improved. It is rather hard to interpret it."

Response:

Fig. S4 (draper protein trap-Gal4 driven GFP-RFP reporter expression) and S5 (intronic STAT92E binding site of the draper gene driven GFP-RFP reporter expression) show similar results:

drpr is already expressed in wing nerve glia before injury, which is in line with Draper's crucial role in the injury response as Draper-mediated glial signaling triggers glial reactivity both in the brain and the wing. This has been added to the Discussion. We have added new data, also mentioned in response to comment R2/6, that also show no major changes in drpr transcript and protein levels between injured and uninjured wing material (Fig. S5D-F).

Minor comments:

R2/11. „Rubicon is also a negative regulator of autophagy (doi:10.1038/s41598-023-44203-6). in (Fig2 B, D) we have a higher GFP intensity in both uninjured and injured, and the difference between Injured/uninjured is less significant compared to control. It is possible that Rubicon KD causes more autophagy leading to a higher activation of Stat92E even in control. I wouldn't take the results as a proof of canonical autophagy implication and not LC3-associated phagocytosis"

Response:

Loss of Rubicon could indeed potentially remove more Su(var)2-10 via increased autophagy, leading to higher Stat92E activity. However, there is no statistically significant difference between uninjured control and Rubicon knockdown and injured control and Rubicon knockdown, respectively, in the data used for Fig. 2D (p=0.6975 and >0.9999 for each comparison) (please see Fig.3 for Reviewers below). We are puzzled by the statement that the reviewer „wouldn't take the results as a proof of canonical autophagy implication and not LC3-associated phagocytosis". We analyzed ***Rubicon*** *as a factor critical for LAP and its* ***deficiency does not prevent Stat transcriptional activity*** *following injury* ***unlike the loss of Atg8a, Atg16, Atg13 and Atg5****. We gained further support for this result with a mutant of Atg16 with part of the WD40 domain deleted. Atg16 WD40 repeats are critical for LAP but not for autophagy.*[18,28,29]*. 10xStat92E enhancer-GFP response was normal after injury in this LAP-specific mutant (Fig. 2E,F) excluding the role of LAP in Stat92E-dependent transcription.*

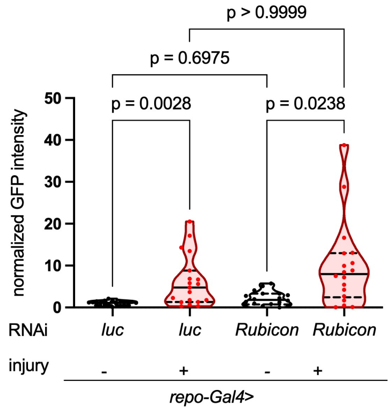

Figure 3 for Reviewers*. – related to Figure 2D****.*** *Quantitative analysis of 10xStat92E enhancer-GFP activation. Kruskal-Wallis test with Dunn's multiple comparison's test. n= 19, 19, 18, 18.*

R2/12. „The rationale for using both repoGal4 and repoGS is unclear. If, as mentioned, the goal is to avoid developmental defects, repoGS should be consistently used. Especially I don't understand how both were utilized to knock down the same genes, such as Atg16"

Response:

We had to use repoGS (a drug-inducible Gal4 active in glia) because knocking down Su(var)2-10 with repoGal4 resulted in no viable adult progeny. Su(var)2-10 is an essential gene as opposed to most autophagy genes and its absence results in embryonic lethality[26]*. Thus all Su(var)2-10 silencing experiments were done with repoGS. Similarly, Stat92E is involved in various developmental processes and its loss is embryonic lethal. repoGal4 was used for genes generally not having an adverse effect when absent during development*[18] *in the first two figures. In Fig. 4D, we silenced Atg16 by repoGS because it is one of the controls for testing a genetic epistasis between Su(var)2-10 and Atg16. Please note that we see exactly the same phenotype in case of Atg16 knockdown when using either Gal4 version. This has been explained in the revised methods section.*

R2/13. „In the third paragraph of the introduction, I am confused whether Stat92E regulates drpr of the reverse"

Response:

Upon antennal injury, Drpr receptor binding to phagocytic cargo initiates a positive feedback loop in glial cells to promote its own transcription[11]*. Drpr receptor protein regulates Stat92E and AP-1 activity via signal transduction. Stat92E and AP-1, in turn, increases drpr transcription*[10,11,30,31] *that will result in more plasma membrane Drpr protein expression. We have explained this more clearly in the revised Introduction.*

R2/14. „I cannot find the evidence for vir-1 being expressed in glia and target of Gcm in the refences that have been cited."

Response:

We apologize for not explaining this better: vir-1 is called CG5453 in Freeman et al., 2003[32]*. It is listed in Table 1 as a Gcm target since there is no detectable CG5453 expression in a Gcm null mutant, please see below. We have updated the manuscript with this gene name.*

Table 1. Glial and Gcm-Regulated Genes Identified in This Study							
Gene	Wild-Type Expression[a]	Ectopic *gcm*[b]	*gcm*⁻	Expression Class[c]	Predicted Product	Functional Class	Human Sequence Homolog
.....							
CG5453	glia	n.d.	-	S, T	novel	other	--
.....							

Part of Table 1 from Freeman et al., 2003[32].

R2/15. „The presence of a Stat92E binding site on the vir-1 promoter has already bene described in the paper from Imler and collaborators, Nature immunology 2005. Actually, if this site is present in their transgenic line, it would help the authors strengthen the argument that Stat92E has a direct role on vir1 (for which they make a very strong statement in the discussion, with no direct evidence)."

Response:

The evidence that Stat92E may have a direct role in vir-1 transcription in glia comes exactly from the same reporter transgene described by Imler and collaborators in the mentioned paper[33]*. We received this transgenic line from the Imler group and monitored its expression after injury upon depletion of Stat92E (Fig. 3B). It thus contains the studied Stat binding site. This was referenced in the Methods and in all relevant sections of the main text, and we now explicitly state this in the revised text.*

R2/16. „In the Fig S2D, I do not see a lot of GFP+ (Glia) cells. I see more Atg8a in injured 3 dpi regardless of colocalization with glia"

Response:

Fig S2D (now S3E) uses one of the standard assays for autophagic turnover, which we now explain in more detail in the Results section. Basically, the dual tagged GFP::mCherry::Atg8a reporter transgene is expressed in glia, and GFP is quenched in lysosomes after delivery by autophagy while mCherry remains fluorescent. So, in addition to double positive dots (autophagosomes), there are mCherry dots lacking GFP (autolysosomes) if autophagy is functional. All of these dots are in glia but the cell boudaries are not visible.

The images shown are single optical slices. The number of mCherry+ puncta are around 7-8 per field in both uninjured and injured (3 dpi) conditions, but puncta brightness is always variable. Since most mCherry+ puncta were rather bright in the original 3 dpi image, we changed it to a more representative image.

R2/17. „The quantification of the signals is made in a specific region of the wing, I guess throughout the nerve thickness. This could be represented more carefully in a schematic and It would also help defining colocalization in the first figure, by using a transverse section."

Response:

The quantification method is described in Materials and Methods and we have added that quantification was done using single optical slices. The imaged region is depicted in Fig. S1A, where we indicated the rectangular region used in Fiji for image quantification. We added transverse sections of wing nerves as suggested (Fig. S2A,B).

R2/18. „A number of ATG genes are considered in the manuscript, but the rational for using them is not always clear. Showing a schematic would help clarify this. „

Response:

We have added a table showing the different steps of autophagyand where the studied Atg genes/proteins function (now Supplementary Table 1). We also added whether the gene is considered specific for autophagy or can play a role in another process, e.g. LAP. We studied different autophagy genes in line with the assumption that disabling distinct autophagic complexes should produce the same phenotype if this process is indeed autophagy (and not LC3-associated phagocytosis for example).

R2/19. „Fig 7 is not cited and its legend is very short."

Response:

We have now cited Fig 7 and expanded its legend.

R2/20. „Clarify the color coding in Fig S1E"

Response:

We added that red is injured, black is uninjured.

R2/21. „What is the tandem tagged autophagic fly reporter in fig S2D?"

Response:

This is one of the most common tools to study autophagy, please see the updated explanation above at your first question (R2/16) regarding Fig. S2D (now S3E).

R2/22. „Add a schematic on the vir-1 isoforms."

Response:

We have added a schematic showing the vir-1 isoforms in Fig. S6B.

R2/23. „Fig S6B and Fig 5 relate on the levels of Su(var)2-10 upon Atg16 RNAi, but the scale is not the same, why?"

Response:

The scales are different because these two images measure different things. Fig. 5 indeed displays quantification of Su(var)2-10 levels in brain glia. Fig S6B (now S7B) shows quantification of Stat92E-induced GFP reporter levels (as a proxy of Stat92E transcriptional activity) in the wing nerve upon Atg16 knockdown.

Reviewer 3

R3/1. „The claim that the negative regulator of Stat92E signaling is removed by selective autophagy, involving selective autophagy receptors different from/in addition to Ref(2)P/p62 is not convincingly shown. This claim probably needs to be softened."

Response:

We have rephrased this sentence as follows:

„These data suggest that selective autophagy is involved in Stat92E-dependent transcriptional activation in glia."

R3/2. „The reporter that was used (10xSTAT92E-eGFP) is not a dynamic reporter of STAT92E activity. It accumulates in glia and is highly stable. The appropriate reporter to look at dynamic changes would be 10XSTAT92E-dGFP, which has a degradable (unstable) GFP that is required to see dynamic changes even in the CNS. All of the claims about STAT92E regulation use this reporter, so they are questionable."

Response:

10XSTAT92E-dGFP featuring destabilized GFP could be a more appropriate tool for monitoring dynamic changes in transcription when short term - e.g. few hours - changes are investigated. Unfortunately, ***we did not see any expression of 10XSTAT92E-dGFP*** *(we tried 2 different transgenic insertions)* ***in the wing nerve, please see Figure 4 for Reviewers.*** *In the brain, dGFP expression with this reporter is also several times lower than stable GFP, please compare Fig. 4A and B in Doherty et al*[11].

The use of 10xSTAT92E-eGFP to follow dynamic expression changes is justified by many lines of evidence. First, there is no 10xSTAT92E-EGFP expression in uninjured wing nerves (Fig. S1B,C). Injury induces EGFP expression in the wing nerve with a sustained activation from 1 to 3 dpi (days post injury), and the EGFP expression returns to the baseline by 5 dpi (Fig. S1B,C). Second, the initial Stat-dependent upregulation of drpr and the 10XSTAT92E-dGFP signal in the brain both occur in the first 24 hours after injury and are sustained for 72 hours[11] *similar to our results with 10xSTAT92E-EGFP in the wing (Fig. S1B,C). These results indicate that the dynamics of 10xSTAT92E-EGFP expression allows monitoring changes in Stat-dependent transcription occurring over days.*

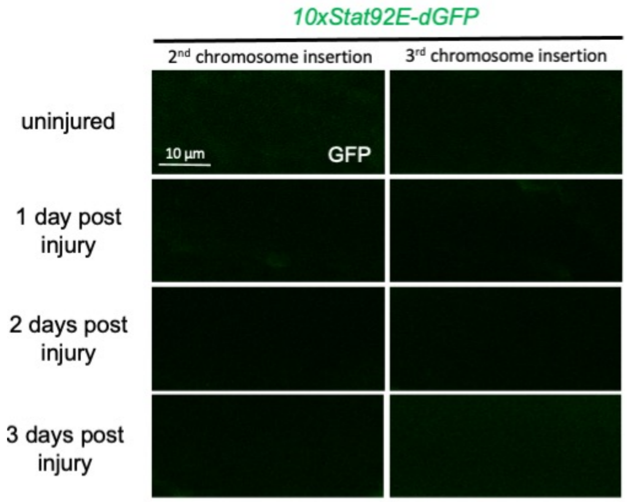

Fig. 4 for Reviewers. Lack of *10XSTAT92E-dGFP* signal in the wing nerve from two independent insertions of the same transgene at the indicated time points after wing injury.

R3/3. „The claim that glial drpr is not upregulated by wing injury and drpr accumulation is not apparently a prerequisite for efficient debris processing within the wing is weak. First, they did not stain for Draper using antibodies, rather they used expression constructs. Dee7 is a promoter that was found to be injury activated in the CNS (were they able to replicate that result? **I did not receive the supplemental data**), but it might not be the crucial regulator in the periphery. The MIMIC line that was converted is better, but might not represent the full spectrum of regulatory events at the draper locus. Finally, they never actually test for endogenous RNA changes, or use the antibody on westerns. Their lack of evidence is not as compelling as it could be."

Response:

*The **original Supplemental Material already provides answers for this and subsequent questions of Reviewer 3**. We deposited the Supplemental Material to bioRxiv at the time of the first Review Commons submission and it was/is available at https://www.biorxiv.org/content/10.1101/2024.08.28.610109v2.supplementary-material.*

***Figs. S4 and S5** show (using two different drpr reporters for its transcriptional regulation) that drpr expression does not change much in the wing after nerve injury, unlike in the brain.*

We did indeed replicate that dee7-Gal4 expression is induced in the brain after antennal injury using UAS- TransTimer (Fig. S5A). In contrast, wing cell nuclei already show expression of both fluorescent proteins in uninjured conditions, and RFP+ nucleus numbers do no change after wing injury (Fig. S5B, C). drpr-Gal4 was generated by conversion of a MiMIC gene trap element

into a Gal4 that traps all transcripts. drpr^{MI07659} is in an intron that is common in all drpr isoforms so it should capture the regulation of all transcript isoforms.

We have further analyzed drpr expression via independent methods during the revision: qPCR amplification of a common region of drpr transcripts, and western blot with anti-Drpr antibody to compare injured and uninjured wing material. We did not observe significant changes in drpr transcript and protein levels (Fig. S5D-F).

Unfortunately, immunostaining of the adult wing is not feasible because antibodies do not penetrate the thick chitin-based cuticle and wax cover of the wing.

R3/4. „The authors claim autophagy contributes to glial reactive states in part by acting on JAK-STAT pathway via regulation of Stat92E. They did not investigate other potential STAT92E targets. Does Atg16 knockdown alter STAT92E expression? Apparently Vir1 is still upregulated in the absence of Atg16 following injury, but they don't show STAT92E changes."

Response:

We did investigate other potential STAT92E targets besides vir-1. This is referred to in the text as „immunity-related gene reporters" and it again can be ***found in the Supplemental Material (Supplementary Table 2)****. None of these genes showed glia-specific upregulation following injury.*

We investigated STAT92E expression with the STAT92E::GFP::FLAG protein-protein fusion transgene after disrupting autophagy as also suggested by Reviewer 2. Please see our detailed answer to the first comment of Reviewer 2.

We do not agree with the comment that „Vir1 is still upregulated in the absence of Atg16 following injury" because Fig. 3F,G show that lack of Atg16 abolishes the upregulation of the vir-1 reporter: the change from uninjured to injured becomes statistically not significant and the mean GFP intensities are practically identical.

R3/5. „The authors claim Su(var)2-10 is an autophagic cargo. They should better characterize Su(var)2-10 degradation and its regulation, and image quality needs to be improved (better images, merged examples, and clearer indication of what they are highlighting. There are many arrows in figures that I don't know what they are pointing to. Much of the labeling in Fig 1 (and others) looks like axons. Could TRE-GFP be turned on in neurons? How did they discriminate?"

Response:

As also explained to Reviewer 1's last comment, we have carried out experiments to address whether SUMOylated Su(var)2-10 binds Atg8a directly. Please see our detailed response there. In brief, we observed no interaction in vitro between either SUMOylated or unmodified GST-Su(var)2-

10 and HA-Atg8a. Therefore we conclude that other protein(s) likely bridge the interaction between Atg8a and Su(var)2-10.

We further improved image quality in Fig. S2 and we incorporated new images in Fig. 2E, the entire Fig. 6, S2, S3B and S7. Merged images were missing only in Fig 5, which we have included in the current version. Arrows and arrowheads were used as described in Figure legends, but instead of those, we now clearly label the epithelium and we outlined the region of wing nerve glia in all images.

Please see our response to the first minor comment of Reviewer 1 regarding the expression of reporters in wing tissues. In short, we find that TRE-GFP is not upregulated in neurons.

R3/6. „The authors claim interaction of Su(var)2-10 with Atg8a in the nucleus and cytoplasm can trigger autophagic breakdown, involving Su(var)2-10 SUMOylation. The paper would benefit from showing direct SUMOylation of Su(var)2-10 after injury. Is there any way to examine this in vivo?"

Response:

We have tested direct SUMOylation of Su(var)2-10 in vivo using a recently described method by Andreev et al., 2022[34]*. Briefly, FLAG-GFP-Smt3 (SUMO) is expressed under SUMO transcriptional regulation in transgenic flies. We immunoprecipitated FLAG-GFP-SUMO with GFPTrap beads from lysates of heads subjected to traumatic brain injury that results in glial reactivity*[18]*, and also from uninjured head lysates. We washed the beads in denaturing conditions. As seen below in Figure 5 for Reviewers, although free SUMO as well as SUMOylated proteins are enriched in precipitates, unfortunately we could not observe Su(var)2-10 SUMOylation in vivo. Of note, Su(var)2-10 self-SUMOylation has only been shown in vitro so far* [12,26]*, so probably its endogenous levels are low.*

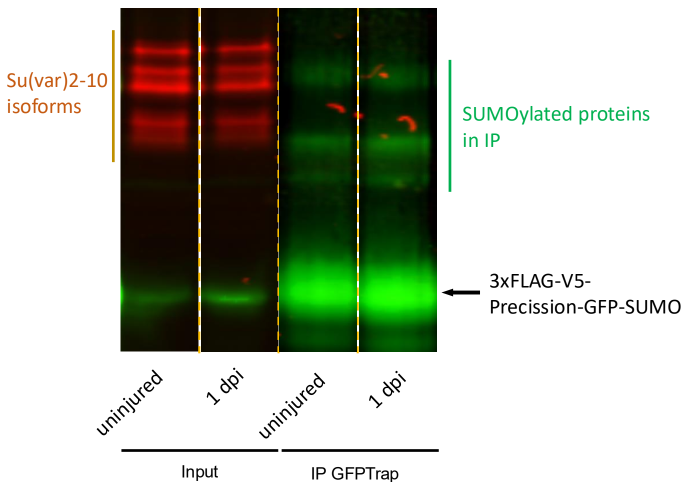

Figure 5 for Reviewers. SUMOylated proteins from FLAG-GFP-SUMO-expressing heads were precipitated by GFPTrap beads in denaturing conditions and blotted for Su(var)2-10. Su(var)2-10 cannot be detected in precipitates (IP GFPTrap).

R3/7. „The authors state in discussion "we find that draper is highly expressed in wing nerve glia already in uninjured conditions and it is not further induced by wing transection - indicating high phagocytic capacity in wing glia ... axon debris clearance takes substantially longer in the wing nerve than in antennal lobe glomeruli, thus draper levels may not readily predict actual phagocytic activity in glia". However, they never actually assess this in their experiments. All the conclusions about Draper are made from promoter fusions of integrated reporters, which are imperfect. This conclusion cannot be made."

Response:

As described in our response to R3/3, we have further analyzed drpr expression via independent methods during the revision: qPCR amplification of a common region of drpr transcripts, and western blot with anti-Drpr antibody on wing material. Briefly, we did not observe major changes in drpr transcript and protein levels between injured and uninjured states (Fig. S5D-F). Nevertheless, we deleted this part criticized by the reviewer from the Discussion because these are not important for the main message of our manuscript.

R3/8. „Both STAT92E and Jun are activated by a stress response. Could this be a stress response to disrupting autophagy that is somehow enhance by injury?"

Response:

Stress responses are indeed relayed by AP-1 and STAT signaling, and impaired autophagy could be a source of stress. We would like to emphasize, though, that the main finding of our manuscript is that ***disrupting autophagy suppresses STAT-dependent transcription****. Autophagy inhibition does not increase STAT signaling in uninjured wing nerves and while control flies upregulate STAT activity upon injury, autophagy-deficient animals fail to do so (Fig. 1). Thus,* ***STAT signaling is not activated by loss of autophagy – it is activated by injury (that is the stress) and STAT activation requires autophagy in this setting.***

R3/9. „Minor:
I don't think that "glially" is a word."

Response:

Online dictionaries such as Wiktionary list glially as a word, and many scientific articles use it: https://doi.org/10.1016/j.conb.2022.102653, https://doi.org/10.1016/j.yexcr.2013.08.016, https://doi.org/10.1016/j.jpain.2006.04.001, to give some examples.

We nonetheless refrain from using it in the updated text.

References

1. Neukomm, L.J., Burdett, T.C., Gonzalez, M.A., Züchner, S., and Freeman, M.R. (2014). Rapid in vivo forward genetic approach for identifying axon death genes in Drosophila. Proc National Acad Sci *111*, 9965–9970. https://doi.org/10.1073/pnas.1406230111.

2. Giangrande, A., Murray, M.A., and Palka, J. (1993). Development and organization of glial cells in the peripheral nervous system of Drosophila melanogaster. Development *117*, 895–904. https://doi.org/10.1242/dev.117.3.895.

3. Stork, T., Engelen, D., Krudewig, A., Silies, M., Bainton, R.J., and Klämbt, C. (2008). Organization and Function of the Blood–Brain Barrier in Drosophila. J. Neurosci. *28*, 587–597. https://doi.org/10.1523/jneurosci.4367-07.2008.

4. Figueras-Novoa, C., Timimi, L., Marcassa, E., Ulferts, R., and Beale, R. (2024). Conjugation of ATG8s to single membranes at a glance. J. Cell Sci. *137*, jcs261031. https://doi.org/10.1242/jcs.261031.

5. Galluzzi, L., and Green, D.R. (2019). Autophagy-Independent Functions of the Autophagy Machinery. Cell *177*, 1682–1699. https://doi.org/10.1016/j.cell.2019.05.026.

6. Nieto-Torres, J.L., Leidal, A.M., Debnath, J., and Hansen, M. (2021). Beyond Autophagy: The Expanding Roles of ATG8 Proteins. Trends Biochem Sci *46*, 673–686. https://doi.org/10.1016/j.tibs.2021.01.004.

7. Huang, Z., Kingsolver, M.B., Avadhanula, V., and Hardy, R.W. (2013). An Antiviral Role for Antimicrobial Peptides during the Arthropod Response to Alphavirus Replication. J. Virol. *87*, 4272–4280. https://doi.org/10.1128/jvi.03360-12.

8. Purice, M.D., Ray, A., Münzel, E.J., Pope, B.J., Park, D.J., Speese, S.D., and Logan, M.A. (2017). A novel Drosophila injury model reveals severed axons are cleared through a Draper/MMP-1 signaling cascade. Elife *6*, e23611. https://doi.org/10.7554/elife.23611.

9. Alphen, B. van, Stewart, S., Iwanaszko, M., Xu, F., Li, K., Rozenfeld, S., Ramakrishnan, A., Itoh, T.Q., Sisobhan, S., Qin, Z., et al. (2022). Glial immune-related pathways mediate effects of closed

head traumatic brain injury on behavior and lethality in Drosophila. Plos Biol *20*, e3001456. https://doi.org/10.1371/journal.pbio.3001456.

10. MacDonald, J.M., Beach, M.G., Porpiglia, E., Sheehan, A.E., Watts, R.J., and Freeman, M.R. (2006). The Drosophila Cell Corpse Engulfment Receptor Draper Mediates Glial Clearance of Severed Axons. Neuron *50*, 869–881. https://doi.org/10.1016/j.neuron.2006.04.028.

11. Doherty, J., Sheehan, A.E., Bradshaw, R., Fox, A.N., Lu, T.-Y., and Freeman, M.R. (2014). PI3K Signaling and Stat92E Converge to Modulate Glial Responsiveness to Axonal Injury. PLoS Biol *12*, e1001985. https://doi.org/10.1371/journal.pbio.1001985.

12. Bence, M., Jankovics, F., Kristó, I., Gyetvai, Á., Vértessy, B.G., and Erdélyi, M. (2024). Direct interaction of Su(var)2-10 via the SIM-binding site of the Piwi protein is required for transposon silencing in Drosophila melanogaster. FEBS J. *291*, 1759–1779. https://doi.org/10.1111/febs.17073.

13. Betz, A., Lampen, N., Martinek, S., Young, M.W., and Darnell, J.E. (2001). A Drosophila PIAS homologue negatively regulates stat92E. Proc. Natl. Acad. Sci. *98*, 9563–9568. https://doi.org/10.1073/pnas.171302098.

14. Liu, B., Liao, J., Rao, X., Kushner, S.A., Chung, C.D., Chang, D.D., and Shuai, K. (1998). Inhibition of Stat1-mediated gene activation by PIAS1. Proc. Natl. Acad. Sci. *95*, 10626–10631. https://doi.org/10.1073/pnas.95.18.10626.

15. Bach, E.A., Ekas, L.A., Ayala-Camargo, A., Flaherty, M.S., Lee, H., Perrimon, N., and Baeg, G.-H. (2007). GFP reporters detect the activation of the Drosophila JAK/STAT pathway in vivo. Gene Expr Patterns *7*, 323–331. https://doi.org/10.1016/j.modgep.2006.08.003.

16. Kroschwald, S., Maharana, S., and Simon, A. (2017). Hexanediol: a chemical probe to investigate the material properties of membrane-less compartments. Matters. https://doi.org/10.19185/matters.201702000010.

17. Hu, X., li, J., Fu, M., Zhao, X., and Wang, W. (2021). The JAK/STAT signaling pathway: from bench to clinic. Signal Transduct. Target. Ther. *6*, 402. https://doi.org/10.1038/s41392-021-00791-1.

18. Szabó, Á., Vincze, V., Chhatre, A.S., Jipa, A., Bognár, S., Varga, K.E., Banik, P., Harmatos-Ürmösi, A., Neukomm, L.J., and Juhász, G. (2023). LC3-associated phagocytosis promotes glial degradation of axon debris after injury in Drosophila models. Nat. Commun. *14*, 3077. https://doi.org/10.1038/s41467-023-38755-4.

19. Goodall, E.A., Kraus, F., and Harper, J.W. (2022). Mechanisms underlying ubiquitin-driven selective mitochondrial and bacterial autophagy. Mol. Cell *82*, 1501–1513. https://doi.org/10.1016/j.molcel.2022.03.012.

20. Zhang, T., Yang, H., Zhou, Z., Bai, Y., Wang, J., and Wang, W. (2022). Crosstalk between SUMOylation and ubiquitylation controls DNA end resection by maintaining MRE11 homeostasis on chromatin. Nat. Commun. *13*, 5133. https://doi.org/10.1038/s41467-022-32920-x.

21. Chen, Z., Zhang, Y., Guan, Q., Zhang, H., Luo, J., Li, J., Wei, W., Xu, X., Liao, L., Wong, J., et al. (2021). Linking nuclear matrix–localized PIAS1 to chromatin SUMOylation via direct binding of histones H3 and H2A.Z. J. Biol. Chem. *297*, 101200. https://doi.org/10.1016/j.jbc.2021.101200.

22. Brown, J.R., Conn, K.L., Wasson, P., Charman, M., Tong, L., Grant, K., McFarlane, S., and Boutell, C. (2016). SUMO Ligase Protein Inhibitor of Activated STAT1 (PIAS1) Is a Constituent Promyelocytic Leukemia Nuclear Body Protein That Contributes to the Intrinsic Antiviral Immune Response to Herpes Simplex Virus 1. J. Virol. *90*, 5939–5952. https://doi.org/10.1128/jvi.00426-16.

23. Gutierrez-Morton, E., and Wang, Y. (2024). The role of SUMOylation in biomolecular condensate dynamics and protein localization. Cell Insight *3*, 100199. https://doi.org/10.1016/j.cellin.2024.100199.

24. Jacomin, A.-C., Petridi, S., Monaco, M.D., Bhujabal, Z., Jain, A., Mulakkal, N.C., Palara, A., Powell, E.L., Chung, B., Zampronio, C., et al. (2020). Regulation of Expression of Autophagy Genes by Atg8a-Interacting Partners Sequoia, YL-1, and Sir2 in Drosophila. Cell Reports *31*, 107695. https://doi.org/10.1016/j.celrep.2020.107695.

25. Maimon, I., Popliker, M., and Gilboa, L. (2014). Without children is required for Stat-mediated zfh1 transcription and for germline stem cell differentiation. Development *141*, 2602–2610. https://doi.org/10.1242/dev.109611.

26. Ninova, M., Chen, Y.-C.A., Godneeva, B., Rogers, A.K., Luo, Y., Tóth, K.F., and Aravin, A.A. (2020). Su(var)2-10 and the SUMO Pathway Link piRNA-Guided Target Recognition to Chromatin Silencing. Mol. Cell *77*, 556-570.e6. https://doi.org/10.1016/j.molcel.2019.11.012.

27. Pircs, K., Nagy, P., Varga, A., Venkei, Z., Erdi, B., Hegedus, K., and Juhasz, G. (2012). Advantages and Limitations of Different p62-Based Assays for Estimating Autophagic Activity in Drosophila. PLoS ONE *7*, e44214. https://doi.org/10.1371/journal.pone.0044214.

28. Fletcher, K., Ulferts, R., Jacquin, E., Veith, T., Gammoh, N., Arasteh, J.M., Mayer, U., Carding, S.R., Wileman, T., Beale, R., et al. (2018). The WD40 domain of ATG16L1 is required for its non-canonical role in lipidation of LC3 at single membranes. EMBO J *37*, e97840. https://doi.org/10.15252/embj.201797840.

29. Rai, S., Arasteh, M., Jefferson, M., Pearson, T., Wang, Y., Zhang, W., Bicsak, B., Divekar, D., Powell, P.P., Nauman, R., et al. (2018). The ATG5-binding and coiled coil domains of ATG16L1 maintain autophagy and tissue homeostasis in mice independently of the WD domain required for

LC3-associated phagocytosis. Autophagy *15*, 1–14. https://doi.org/10.1080/15548627.2018.1534507.

30. Logan, M.A., Hackett, R., Doherty, J., Sheehan, A., Speese, S.D., and Freeman, M.R. (2012). Negative regulation of glial engulfment activity by Draper terminates glial responses to axon injury. Nat. Neurosci. *15*, 722–730. https://doi.org/10.1038/nn.3066.

31. MacDonald, J.M., Doherty, J., Hackett, R., and Freeman, M.R. (2013). The c-Jun kinase signaling cascade promotes glial engulfment activity through activation of draper and phagocytic function. Cell Death Differ *20*, 1140–1148. https://doi.org/10.1038/cdd.2013.30.

32. Freeman, M.R., Delrow, J., Kim, J., Johnson, E., and Doe, C.Q. (2003). Unwrapping Glial Biology Gcm Target Genes Regulating Glial Development, Diversification, and Function. Neuron *38*, 567–580. https://doi.org/10.1016/s0896-6273(03)00289-7.

33. Dostert, C., Jouanguy, E., Irving, P., Troxler, L., Galiana-Arnoux, D., Hetru, C., Hoffmann, J.A., and Imler, J.-L. (2005). The Jak-STAT signaling pathway is required but not sufficient for the antiviral response of drosophila. Nat. Immunol. *6*, 946–953. https://doi.org/10.1038/ni1237.

34. Andreev, V.I., Yu, C., Wang, J., Schnabl, J., Tirian, L., Gehre, M., Handler, D., Duchek, P., Novatchkova, M., Baumgartner, L., et al. (2022). Panoramix SUMOylation on chromatin connects the piRNA pathway to the cellular heterochromatin machinery. Nat. Struct. Mol. Biol. *29*, 130–142. https://doi.org/10.1038/s41594-022-00721-x.

November 20, 2025

RE: Life Science Alliance Manuscript #LSA-2025-03375-TR

Dr. Áron Szabó
HUN-REN Szegedi Biológiai Kutatóközpont
Institute of Genetics
Temesvári krt 62
Szeged 6726
Hungary

Dear Dr. Szabó,

Thank you for submitting your revised manuscript entitled "Selective autophagy fine-tunes Stat92E activity by degrading Su(var)2-10/PIAS in Drosophila glia". We returned your manuscript to the original reviewers from Review Commons. Regrettably only Reviewer 1 was available to evaluate this revised work. Please consider discussing potential benefits of using a dGFP reporter as was sought by Reviewer 3 in point 2 and justifying your use of the eGFP reporter based on the kinetics of your assays. As Reviewer 1 is satisfied, and the editors have judged that the remaining reviewer points have been addressed, we would be happy to publish your paper in Life Science Alliance pending final revisions necessary to meet our formatting guidelines.

Along with points mentioned below, please tend to the following:
-Please be sure that the authorship listing and order is correct.
-Please add the X and Bluesky handles of your host institute/organization, as well as your own and/or one of the authors, in our system.
-LSA does not permit citation of "data not shown," "manuscript in preparation," "manuscript submitted," etc., in any section of the manuscript. Please include the rebuttal Figure 2 (showing no interaction between Su(var)2-10 and Atg8a) to support the statement on Line 407.
-We discourage making data available upon request rather than having it publicly available, but if there is no alternative for privacy or other reasons, describe what the data are and why they are not public, whom to contact (with a public email address), and conditions for re-use.
-Please clearly mark panel B in Figure 4.
-We encourage you to revise the figure legends for Figure S1 such that the figure panels are introduced in alphabetical order.
-Please indicate the scale bar size in the Legend for Figure S2A.
-There is a callout for figure S8D, and the figure and its legend don't have this panel. Please correct accordingly.
-Please consider using Figure 8 as a graphical abstract for this work, noting that these are not included in pdf files but are only online. To make this change please remove this as a figure file and upload this image instead with the file type "graphical abstract."

If you are planning a press release on your work, please inform us immediately to allow informing our production team and scheduling a release date.

LSA now encourages authors to provide a 30-60 second video where the study is briefly explained. We will use these videos on social media to promote the published paper and the presenting author (for examples, see https://docs.google.com/document/d/1-UWCfbE4pGcDdcgzcmiuJl2XMBJnxKYeqRvLLrLSo8s/edit?usp=sharing). Corresponding or first-authors are welcome to submit the video. Please submit only one video per manuscript. The video can be emailed to contact@life-science-alliance.org

To upload the final version of your manuscript, please log in to your account: https://lsa.msubmit.net/cgi-bin/main.plex
You will be guided to complete the submission of your revised manuscript and to fill in all necessary information. Please get in touch in case you do not know or remember your login name.

To avoid unnecessary delays in the acceptance and publication of your paper, please read the following information carefully.

A. FINAL FILES:

These items are required for acceptance.

-- An editable version of the final text (.DOC or .DOCX) is needed for copyediting (no PDFs).

-- High-resolution figure, supplementary figure and video files uploaded as individual files: See our detailed guidelines for

preparing your production-ready images, https://www.life-science-alliance.org/authors

-- Summary blurb (enter in submission system): A short text summarizing in a single sentence the study (max. 200 characters including spaces). This text is used in conjunction with the titles of papers, hence should be informative and complementary to the title. It should describe the context and significance of the findings for a general readership; it should be written in the present tense and refer to the work in the third person. Author names should not be mentioned.

B. MANUSCRIPT ORGANIZATION AND FORMATTING:

Full guidelines are available on our Instructions for Authors page, https://www.life-science-alliance.org/authors

We encourage our authors to provide original source data, particularly uncropped/-processed electrophoretic blots and spreadsheets for the main figures of the manuscript. If you would like to add source data, we would welcome one PDF/Excel-file per figure for this information. These files will be linked as supplementary "Source Data" files.

Submission of a paper that does not conform to Life Science Alliance guidelines will delay the acceptance of your manuscript.

It is Life Science Alliance policy that if requested, original data images must be made available to the editors. Failure to provide original images upon request will result in unavoidable delays in publication. Please ensure that you have access to all original data images prior to final submission.

The license to publish form must be signed before your manuscript can be sent to production. A link to the license to publish form will be available to the corresponding author only. Please take a moment to check your funder requirements.

Reviews, decision letters, and point-by-point responses associated with peer-review at Life Science Alliance will be published, alongside the manuscript. If you do want to opt out of having the reviewer reports and your point-by-point responses displayed, please let us know immediately.

Thank you for your attention to these final processing requirements. Please revise and format the manuscript and upload materials as soon as you are able.

Thank you for this interesting contribution, we look forward to publishing your paper in Life Science Alliance.

Sincerely,

Tim Fessenden
Scientific Editor
Life Science Alliance

Reviewer #1 (Comments to the Authors (Required)):

This is a revised version of a manuscript documenting the effect of autophagy on the regulation of SSAT92E expression via the degradation of the Su(var)2-10/PIAS regulatory protein.

In this revised version the authors have appropriately addressed all concerns raised by reviewers of the initial manuscript and substantially improved the manuscript which is now ready for publication.

December 3, 2025

RE: Life Science Alliance Manuscript #LSA-2025-03375-TRR

Dr. Áron Szabó
HUN-REN Szegedi Biológiai Kutatóközpont
Institute of Genetics
Temesvári krt 62
Szeged 6726
Hungary

Dear Dr. Szabó,

Thank you for submitting your Research Article entitled "Selective autophagy fine-tunes Stat92E activity by degrading Su(var)2-10/PIAS in Drosophila glia". It is a pleasure to let you know that your manuscript is now accepted for publication in Life Science Alliance. Congratulations on this interesting work.

The final published version of your manuscript will be deposited by us to PubMed Central upon publication. We have noted your request to delay publication until January 2025.

Your manuscript will now progress through copyediting and proofing. During proofing please ensure you make a minor correction to the manuscript text: LSA does not have a "Materials Availability" section although we certainly encourage authors to share materials of their studies as requested. Please include the text for this section in the above "Data Availability" section.

Reviews, decision letters, and point-by-point responses associated with peer-review at Life Science Alliance will be published, alongside the manuscript. If you do want to opt out of having the reviewer reports and your point-by-point responses displayed, please let us know immediately.

IMPORTANT: If you will be unreachable at any time, please provide us with the email address of an alternate author. Failure to respond to routine queries may lead to unavoidable delays in publication.

Scheduling details will be available from our production department. You will receive proofs shortly before the publication date. Only essential corrections can be made at the proof stage so if there are any minor final changes you wish to make to the manuscript, please let the journal office know now.

DISTRIBUTION OF MATERIALS:
Authors are required to distribute freely any materials used in experiments published in Life Science Alliance. Authors are encouraged to deposit materials used in their studies to the appropriate repositories for distribution to researchers.

You can contact the journal office with any questions, contact@life-science-alliance.org

Again, congratulations on a very nice paper. I hope you found the review process to be constructive and are pleased with how the manuscript was handled editorially. We look forward to future exciting submissions from your lab.

Sincerely,

Tim Fessenden
Scientific Editor
Life Science Alliance
